# Worldwide Alien Invasion: A Methodological Approach to Forecast the Potential Spread of a Highly Invasive Pollinator

**DOI:** 10.1371/journal.pone.0148295

**Published:** 2016-02-16

**Authors:** André L. Acosta, Tereza C. Giannini, Vera L. Imperatriz-Fonseca, Antonio M. Saraiva

**Affiliations:** 1 Department of Ecology, Bioscience Institute, Universidade de São Paulo, Rua do Matão, travessa 14, n. 321, 05508–090, São Paulo, São Paulo, Brazil; 2 Vale Institute of Technology—Sustainable Development, Rua Boaventura da Silva, n. 955, 66055–090, Belém, Pará, Brazil; 3 Department of Computing and Digital Systems Engineering, Polytechnic School, Universidade de São Paulo, Av. Prof. Luciano Gualberto, n. 380, 05508–970, São Paulo, São Paulo, Brazil; 4 Research Center on Biodiversity and Computing–BioComp, Av. Prof. Luciano Gualberto, travessa 3, n.158, 05508–900, São Paulo Capital, São Paulo State, Brazil; Monash University, AUSTRALIA

## Abstract

The ecological impacts of alien species invasion are a major threat to global biodiversity. The increasing number of invasion events by alien species and the high cost and difficulty of eradicating invasive species once established require the development of new methods and tools for predicting the most susceptible areas to invasion. Invasive pollinators pose serious threats to biodiversity and human activity due to their close relationship with many plants (including crop species) and high potential competitiveness for resources with native pollinators. Although at an early stage of expansion, the bumblebee species *Bombus terrestris* is becoming a representative case of pollinator invasion at a global scale, particularly given its high velocity of invasive spread and the increasing number of reports of its impacts on native bees and crops in many countries. We present here a methodological framework of habitat suitability modeling that integrates new approaches for detecting habitats that are susceptible to *Bombus terrestris* invasion at a global scale. Our approach did not include reported invaded locations in the modeling procedure; instead, those locations were used exclusively to evaluate the accuracy of the models in predicting suitability over regions already invaded. Moreover, a new and more intuitive approach was developed to select the models and evaluate different algorithms based on their performance and predictive convergence. Finally, we present a comprehensive global map of susceptibility to *Bombus terrestris* invasion that highlights priority areas for monitoring.

## Introduction

The ecological impacts of species invasion area major threat to global biodiversity [1, 2], with widespread effects on humanity [3, 4]. An alien invasive species is defined as a taxon introduced outside its native range, either deliberately or accidentally, presenting a high growth rate and fast range expansion, with noticeable environmental impacts [5–8].

The number of invasion events by alien speciesis rapidly increasing around the globe [1, 9]. Thus, the development and application of new methods and tools that allows predict the most susceptible areas to invasion are needed.

Species distribution modeling (SDM) has been applied to forecast the potential occupancy of a wide range of invasive species. Examples include plants (e.g., [10–12]), insects (e.g., [13–16]), mollusks (e.g., [17]) and amphibians (e.g., [18, 19]).

According to Lavergne *et al*.(2010) [20], SDMs have been largely influenced by Hutchinson’s concept of the ecological niche [21]. Currently, this type of ecological modeling is also considered able to estimate habitat suitability, as it uses reported occurrences to identify other places with similar suitable conditions [22]. In this work, we choose Habitat Suitability Modeling (HSM) as a standard denomination of an analytical procedure that encompasses methods and concepts described by other researchers and authors as Species Distribution Modeling (SDM) or Ecological Niche Modeling (ENM).

Abiotic factors are considered the initial environmental barrier that an invasive species must overcome when it is introduced or when it invades a non-native environment [23–27] sincemost individuals cannot survive on an environment where abiotic factors exceed their physiological limits [22, 27]. At large scale, climatic factors can be considered among the most influential types of abiotic factors that limit or, in some cases, promote the expansion process of an invasive species [11, 25, 28, 29].

Several HSM methodological approaches have been proposed to improve their application. For example, some studies have compared the performance of different algorithms (e.g., [30]) or sample sizes (e.g., [31]); or examined how to fit models (e.g., [32]); ensemble multiple projections (e.g., [33]); included biotic interactions (e.g., [34]); or evaluated model performance (e.g., [35]). Additionally, some studies have used HSMs to assess information on invasive species at many spatial scales (e.g., [11, 36–39]). However, as far as we know, there is no methodological approach for predicting areas that are susceptible to bee invasion on a worldwide scale.

The bumblebee *Bombus terrestris* (L.)(Hymenoptera: Apidae) may become one of the most representative cases of bee invasion at global scale [40], especially considering its velocity of spread (e.g., 200 Km per year in Chile and Argentina [41]).

*Bombus terrestris*is a eusocial bee with a relatively large and hairy body of high thermoregulatory capacity. This species exhibits a generalist feeding habits, being able to explore a wide range of floral resources. The wild, native distributional range of *Bombus terrestris* covers almost the whole of Europe, mainly the temperate and Mediterranean zones, and encompasses surrounding areas of Asia and Africa [40, 42, 43].

*Bombus terrestris* (hereafter referred to as Bt) is able to perform buzz pollination [40, 44,45], which improves the pollination success of plants with poricidal anthers. Bt provides this important ecosystem service to wild plants and crop species with high economic value such as tomato, pumpkin, eggplant, potato and pepper [44, 46, 47]. Bt is well adapted to artificial conditions, and because of its ease of handling and breeding, colonies have been developed in captivity and commercialized for over 20 years to improve crop pollination, mainly in the greenhouse [40, 47–49].

These Bt´s commercial colonies are delivered to many countries, including some places located outside their natural range. This international trade is reported as the main cause of Bt invasion in Chile, China, Israel, Japan, Mexico, South Africa, South Korea and Taiwan [40–42, 48–58]. Bt is also invasive species in New Zealand and Tasmania (AUS). In New Zealand, Bt was introduced around 1884 for crop pollination purposes and currently is widely spread over the islands [59, 60]. There is no precise information on when Bt was first introduced in Tasmania. Semmens et al., (1993) [61] suggest that Bt individuals were brought to Tasmania from New Zealand in 1992.

Currently, the invasive distribution of Bt is increasing in addition to direct human intervention, by means of its own dispersal capabilities (e.g. [50, 62]). Invasive Bt was reported as presenting some negative effects on native bees; for instance, competing for nesting sites [63], floral resources [64], as a potential vector of exotic diseases and parasites [65, 66] and changing plant-pollinator interactions in non-native environments, impacting crops, native plants and pollinators [67, 68].

Therefore, we consider Bt as a representative case study of an incipient worldwide alien invasion, undergoing rapid expansion in many countries outside its native range.

We present here a methodological framework with new analytical and geospatial strategies to evaluate, select and ensemble the habitat suitability models based on a multi-algorithm approach, aiming to increase the overall predictive accuracy for invasive species studies.

We used the framework to detect susceptible areas to Bt invasion in a worldwide scale and to detect the potential range of invasive spread from already invaded areas. In this work we use exclusively global high-resolution topoclimatic variables in order to provide a method with wide applicability for invasive species and large-scale events of invasions; but the method is not limited to these variables and can be integrated with other bionomic data if necessary.

The global map of susceptibility to Bt invasion delineates areas that should be monitored to avoid new deliberated introduction of colonies and can be used to guide the development of precautionary measures and policies in order to avoid or mitigate future impacts on natural environments and human activities.

## Materials and Methods

### Environmental variables

We obtained 20 layers of environmental topoclimatic data (19 bioclimatic and altitude) from Worldclim [69], with a spatial resolution of 5 minutes of arc (cell size approximately 10 km) over a global range (with the exception of southern latitudes greater than 60°). These layers present data on altitude and annual trends of seasonality, temperature extremes and average precipitation over the last 50 years.

To reduce co-linearity among predictors, we performed a Pearson’s pairwise correlation procedure using R v.3.0.3 [70] and selected those layers with Pearson's correlation coefficients less than 0.75. When two layers were highly correlated, we chose the one least correlated, yielding a total of nine layers: Mean Temperature Diurnal Range, Maximum Temperature of the Warmest Month, Temperature Annual Range, Precipitation of the Wettest Month, Precipitation of the Driest Month, Precipitation Seasonality, Precipitation of the Warmest Quarter, Precipitation of the Coldest Quarter, and Altitude.

We did not include additional environmental variables in the modeling procedure due to the lack of available knowledge about the relationship of Bt with other abiotic and biotic variables in non-native environments. Rather, because climate and altitude have several similar conditions and combinations around the globe, we considered the extrapolation of ecological considerations based solely on topoclimatic variables to be more reliable.

### *Bombus terrestris* data

We used presence records for Bt surveyed from two main sources (for source details, see S1 File): 1) presence data extracted from published literature and 2) presence data obtained from collections and museums and compiled in internet biodiversity databases (mainly from GBIF–Global Biodiversity Information Facility). For the published data, when Bt presence was georeferenced by city or other place name, the geographical coordinates were extracted using the toponyms from the Global Administrative Areas Database [71]. If occurrences were exhibited on a map only, we plotted the map into ArcGIS 10 [72] to estimate the geographical coordinates for each point using the Georeferencing Tool.

The complete dataset was divided into two subsets based on published data (see S1 File): 1) presence within the native Bt range, referred to as *Native Presence* and 2) reported alien invasive presence, referred as *Invasive Presence* (see S1 File). Although some publications have suggested the presence of invasive Bt in China, Israel, Mexico, South Africa, South Korea, and Taiwan, we have found no recorded locations. Thus, we only considered as invaded those places with reports of sightings of individuals or colonies of Bt. Both subsets were plotted in ArcGIS10 [72].

First, we used the sample function and visual inspections to detect unreliable or erroneous records. Any record located in a water body class (e.g., oceans, rivers, lakes) according to GlobCover 2009 land cover (cell size of approximately 0.0028° = ~300 m) [73] was excluded. Furthermore, we excluded presence records from outside the range of the environmental layers selected for modeling or from countries lacking previous reports of native or invasive occurrences.

To reduce statistical overfitting due to a large number of native presence records (over 10,000 raw data records) and to avoid redundancy [74–76], we built a fishnet composed of square cells with the same spatial extent and grid cell size of the environmental layers (~10x10 km). Subsequently, we joined the fishnet with the native Bt presence dataset in ArcGIS. To do so, we treated each respective location as a unique presence, using the centroid of fishnet cells as the geographic coordinate of species presence. This procedure transformed Bt presence into a binary variable; i.e., we attributed a value of 1 to cells with one or more geospatially coincident presence records and a value of 0 to cells without presence records. The same procedure was then conducted with invasive Bt presence records.

### Pseudo-absence datasets

All the modeling algorithms used in this work required the input of pseudo-absences when true absences were not available (in the case of MAXENT, background points) [77–81]. Because we cannot identify the true absence data for Bt, we generated two types of pseudo-absence datasets, as follows.

The first type of pseudo-absence dataset was generated by surveying the GBIF data provider to obtain presence records of *Bombus* species other than Bt, inside countries with reported native Bt presence. This dataset is hereafter referred as BOPA (*Bombus* Other than Bt- Pseudo-Absences). Based on studies of foraging distance in Bt and the maximum distance a worker bee can travel before returning to its nest (e.g., [43, 82, 83]), we determined that Bt workers usually forage close to their nests, generally within 1 km [83]. However, under extreme circumstances (e.g., a scarcity of resources), a worker bee can travel greater distances; the furthest distance reported to date is 9.8 km [82]. We used the largest distance (10 km) as the maximum displacement distance of individual Bt from their nests, and we assumed that most Bt sightings could be positioned within this maximum range during the field surveys, which provided each record in our presence dataset.

Considering the previous assumption, we plotted both the BOPA and native Bt presence datasets into ArcGIS 10 [72], and using the function *select by location*, we detected and excluded those BOPA records located less than 10 km from a Bt presence record. This procedure removed any pseudo-absence record (BOPA) located within the maximum spatial range of each Bt record. The remaining BOPA locations, i.e., field surveys reporting no Bt but reporting other *Bombus* spp., were considered as reliable pseudo-absence locations. As performed previously with the native and invasive dataset, we used the fishnet to remove repeated BOPA locations per grid cell.

The second type of pseudo-absence dataset was randomly generated following two steps. First, in ArcGIS 10 [72], we created a spatial buffer using the geographic locations of both datasets: BOPA and the native presence dataset (**not** the invasive one). In addition, we considered the 10 km distance to define the buffer radius per record for both datasets, and the area jointly covered by all buffers was used as a restriction zone in the next step. Second, we used the function *create random points* of ArcGIS to generate a random pseudo-absence dataset with three spatial constraints. Random points were generated exclusively inside the extent of the climatic layers, outside the restriction zone defined in the previous step, and outside the water bodies class of land cover GlobCover 2009 [73]. Additionally, we prevented the creation of points in repeated locations per grid cell, attributing a minimum distance of 15km between points. The total number of random points was calculated as follows:

$$RPA\;=\;\left[\left(10\;*\;{Native\;Presences}_{\left(total\right)}\right)\;*\;5_{\left(PA\;replications\right)}\right]-\;\left(5_{\left(PA\;replications\right)}\;*\;{BOPA}_{\left(total\right)}\right)$$

Incorporating the obtained results (see below) yields the following:

RPA=[(10*4,209)*5]−(5*3,422)=193,340

The total number of Random Pseudo-Absence Points (RPA) generated (193,340) was randomly fractionated into five subsets without replacement (38,668 per subset), such that each subset held only exclusive locations, with no duplicates. Subsequently, for each of the five random pseudo-absence subsets, we added the BOPA records, totaling 42,090 records per subset. This yielded a total 10 times the number of native presence data points per subset (as recommended by Chefaoui and Lobo, 2008) [84].

### Modeling Procedure

Bt *native presence* was randomly partitioned such that 75% of the data was used for training the model and the remainder (25%) was used for mathematical evaluation using True Skill Statistic (TSS) [85]. This random partitioning was repeated five times to obtain a robust estimate of the algorithms' performance [86].

To generate the habitat suitability models, we used the Biomod2 package version 3.1.48 [80, 81] in R language [70] with all available algorithms in the package (details can be found on S2 File).

To achieve comparability among algorithm results and considering the most frequently used parameters, we maintained the default settings in Biomod2 following the parameters recommended by the authors [80, 81]. The MAXENT algorithm was used in the same way, with default settings recommended by the authors [87], but with a different memory allocation size. These default parameters are described on S2 File.

Twenty-five models were fitted per algorithm (ALGO1 to ALGO10); this was accomplished by combining five native presence partitioning (RUN1 to RUN5) and five pseudo-absence datasets (PA1 to PA5). The same pairwise combination was repeated in each round with each algorithm [ALGO1&RUN1&PA1; ALGO1&RUN1&PA2 (…); ALGO1&RUN2&PA1 (…); ALGO2&RUN1&PA1 (…)]. We obtained 250 models, considering all possible combinations of ALGOs (10), RUNs (5) and PAs (5).

### Model Selection

We developed a sequence of three evaluation criteria to select from the obtained models, as follows:

#### Stage 1

*TSS ≥ 0*.*8*: First, we used a TSS evaluation index (True Skill Statistics; [85]) greater than or equal to |0.8|. We chose TSS instead of AUC (Area Under the Curve of Receiver-Operating Characteristics) because a threshold-dependent measure was necessary to define the spatial cutting point before delineating each respective suitable (binary value = 1) and unsuitable area (binary value = 0) per model. TSS values range between -1 and 1; positive values near one indicate high predictive accuracy. Negative values and zero proximity indicate that the model performs no better than chance, consequently, the models are not useful for detecting habitat suitability [88, 89]. There are no precise threshold TSS scores defined for model evaluation; we chose a more conservative value (TSS ≥ 0.8) than the threshold values frequently used (TSS ≥ 0.75; e.g., [39, 90–92]).

#### Stage 2

*Based on invasive presence hit rate*: In the second stage, we evaluated the accuracy of each remaining model (those with TSS ≥ 0.8) to infer suitability predictions over areas other than the proximal native range of Bt. This method takes into account the rating of the geospatial coincidence of the suitable areas detected by each model (binary value = 1) with the known locations of invasive Bt presence. In this context, the invasive dataset acts as a “validation dataset” for evaluating model accuracy by quantifying the accuracy of suitability detection of each model over the known distribution of invasive Bt.

To calculate this, we used the sensitivity component of TSS metrics. Thus, we calculated the probability of detection or hit rate (HR) according to the formula:

Invasive Hit Rate (IHR) = Hits(binary one) / (Hits(binary one) + Misses(binary zero))

The procedure of intersection between the Stage 1 models and the invasive presence dataset was developed in R [70] using the function *extract* of the Raster package [93].

We assumed that the most accurate models would be those that predicted the records of the validation dataset with higher precision, i.e., the models with higher Invasive Hit Rate (IHR) values. The minimum accuracy threshold for selecting the most precise models was defined by the overall average IHR value, meaning that each individual model that yielded an IHR value equal to or higher than the average IHR of all models was selected to proceed to Stage 3.

The procedure of intersection was replicated using the native Bt presence data, building a dataset used exclusively to compare stage performance in the final evaluation. Each value of this dataset was considered a measure of the Native Hit Rate (NHR).

#### Stage 3

*Convergence of suitability predictions*: The third stage was applied aiming to filter models with statistical biases related to under- and over-fitting. These undesirable statistical effects have to be considered mainly when a massive number of presence data points and wide-extent, high-resolution environmental variables are used. The extensive amount of information incorporated into each environmental layer (worldwide extent: totaling 2,287,025 cells with information per layer) can jeopardizethe specificity component of the TSS evaluation metrics, even when a large number of pseudo-absences per modeling rounds are used. Another important advantage of this criterion is the detection and exclusion of high divergent predictions. Metaphorically, when facing multiple points of view provided by various experts about on a subject, it is usually desirable to consider the opinion shared by the majority rather than the divergent opinion(s) provided by a minority. Therefore, only the models with considerable differences were filtered at this stage, i.e., those strongly diverging in the shape and size of suitable areas detected and differing from a large number of other models generated. We emphasize that the small-scale differences among models are of great importance in a multi-modeling approach; these small differences increase the quality of the result and were not filtered by this procedure.

To evaluate the level of concordance among models, we calculated a similarity index using Pearson´s pairwise correlation coefficients in R [94], as highly correlated model pairs are presumably more similar in terms of habitat suitability and unsuitability prediction. For the models remaining after the previous selection stage, we estimated Pearson´s correlation coefficients between model pairs. For each model, we averaged the coefficients over all paired coefficient values with the other models (except with the model itself; value = 1). These averages (hereafter referred as PCCs—Pearson´s correlation coefficient) were used to rank the similarity of each model in relation to the others. Thus, a model with a high average PCC is more similar in its suitability predictions with the majority of models in the set than models with lower PCCs and vice versa (the lower the PCC average, the less similar).

We predefined an ascending sequence of minimum average PCC thresholds from 0.5 to 1 in increments of 0.01, totaling 51 PCC thresholds. We used this threshold sequence to generate sets of models with average PCC scores equal to or higher than each respective value. For each set generated, we projected an Ensemble Model termed the *Overall Predictions Model* (OPM; description provided in the next topic). Subsequently, we assessed the quality of each generated OPM to correctly predict suitability where the native (NHR) and invasive Bt presence (IHR) records were located. Moreover, we verified the ratio between the number of suitable grid cells predicted and the total number of grid cells per OPM (suitable + unsuitable cells) and termed this ratio the Suitable Cells Ratio (SCR), which was calculated as follows:

$$SCR={OPM}_{i\;(binary\;value\;1\;cells)}\;/\left({OPM}_{i\;\left(binary\;value\;1\;cells\right)}+\;{OPM}_{i\;\left(binary\;value\;0\;cells\right)}\right)$$

We also captured and calculated the minimum, maximum and average TSS values from the set of models that composed each OPM using the TSS values obtained from the Biomod2 output.

### Ensemble forecast

Ensemble forecast models are mathematical methods that combine multiple simulations (forecasts) of a complex system into a unique and more robust result. We developed our ensemble forecast model based on the *committee averaging *method of Biomod2, in which the probabilities of habitat suitability from different models are not averaged but are transformed into binary results [33, 39, 80]. We used each respective model threshold that maximizes both sensitivity and specificity to define the spatial cut-off, before converting each model in binary predictions. This threshold parameter is considered to produce the most accurate results [39, 88, 95]. Another advantage of the committee averaging method is the ease of comparing outputs (binary = 1 = presence; binary = 0 = absence) relative to the raw algorithm outputs (continuous probabilities) that do not necessarily have the same meaning or same range of variation (for further details, see [33] and [39]). Using binary models from each selection criterion (Stages 1, 2 and 3) and the Biomod2 output models, i.e., models without selection (hereinafter referred as Stage 0 models), we developed ensemble forecasts in two steps as follows:

**Step 1)** Agreement Level Ensemble Model (ALM). This model is based on the sum of binary values of each set of models, resulting in maps with geospatial classes ranging from zero (all models agree with the unsuitability of the area) to the total number of geospatial coincidences of suitable habitats detected by all models (a spatialized frequency histogram). Therefore, the class with value = 1 indicates that only one model in the set indicated suitability in the area, the class with value = 2 indicates that two models agreed, and so on. Note that this range is not related to probabilistic or suitability level but to the level of agreement among models in each set. We used ALMs twice. First, they were used to build the next ensemble model type. Second, they were used to build ensembles from each evaluation stage per algorithm, providing a way to compare and evaluate algorithm performance.

**Step 2)** Overall Prediction Ensemble Model (OPM). This model constitutes a binary map that considers every suitable habitat area predicted by the overall models of each set. Essentially, all classes exhibiting values equal to or higher than 1 in the Agreement Level Ensemble Model (previous step) were reclassified as a unique binary value = 1. In contrast, the unsuitable areas retained the binary value = 0. Each OPM was evaluated with respect to its predictive quality, and the selected OPMs were used to build the main ensemble model result, as described below.

### Evaluating and selecting ensemble forecast models

To generate a global map of susceptibility to *Bombus terrestris* invasion, we first selected the most accurate set of models that were, subsequently, combined into a single model using techniques of ensemble forecast. Selection was performed by visual inspection of plotted curves and analysis of changes in four evaluation indices (TSS, Invasive and Native Hit Rates, and Suitable Cells Ratio).We emphasized Invasive Hit Rate (IHR), which compares the obtained model to known invaded areas, a distinctive method of our work.

Among all Overall Prediction Models (OPMs) generated across the predefined Pearson´s Correlation Coefficients (PCC) in Stage 3, we prioritized the selection of those positioned just before marked shifts in the evaluation indices, paying particular attention to IHR changes. When we detected a sequence of changes in the evaluation indices across the PCC range, showing a clear trend, we selected the OPM immediately preceding this sequence.

We also paid attention to the relationship between IHR and the SCR, as well as the relationship of IHR with shifts in the NHR and TSS values (minimum, mean and maximum) across the PCC range. We avoided choosing the last OPMs of the full PCC range because each of the previous OPMs in the range contains the suitable prediction of the next one, and the last OPMs tend to exhibit higher error than previous ones (as detected in the analysis). We selected the minimum possible number of OPMs, considering the highest representativeness of each selection to encompass the main changes across the PCC range.

Once the PCC thresholds were selected, the main ensemble model was produced by the sum of the respective OPMs using the raster package [93] in the R platform [70]. We considered the highest class value resulting from the maximum geospatial coincidence of shared suitability prediction as the "Susceptible at Maximum". To the class value = 1 (only one OPM indicated suitability), we attributed the name "Susceptible". To the intermediate class values, we attributed denominations related to their levels of susceptibility. To the class value = 0 (all OPMs agree with unsuitability of the area), we attributed the denomination of “Very Low Susceptibility or Insusceptible”.

## Results

**Fig 1:**
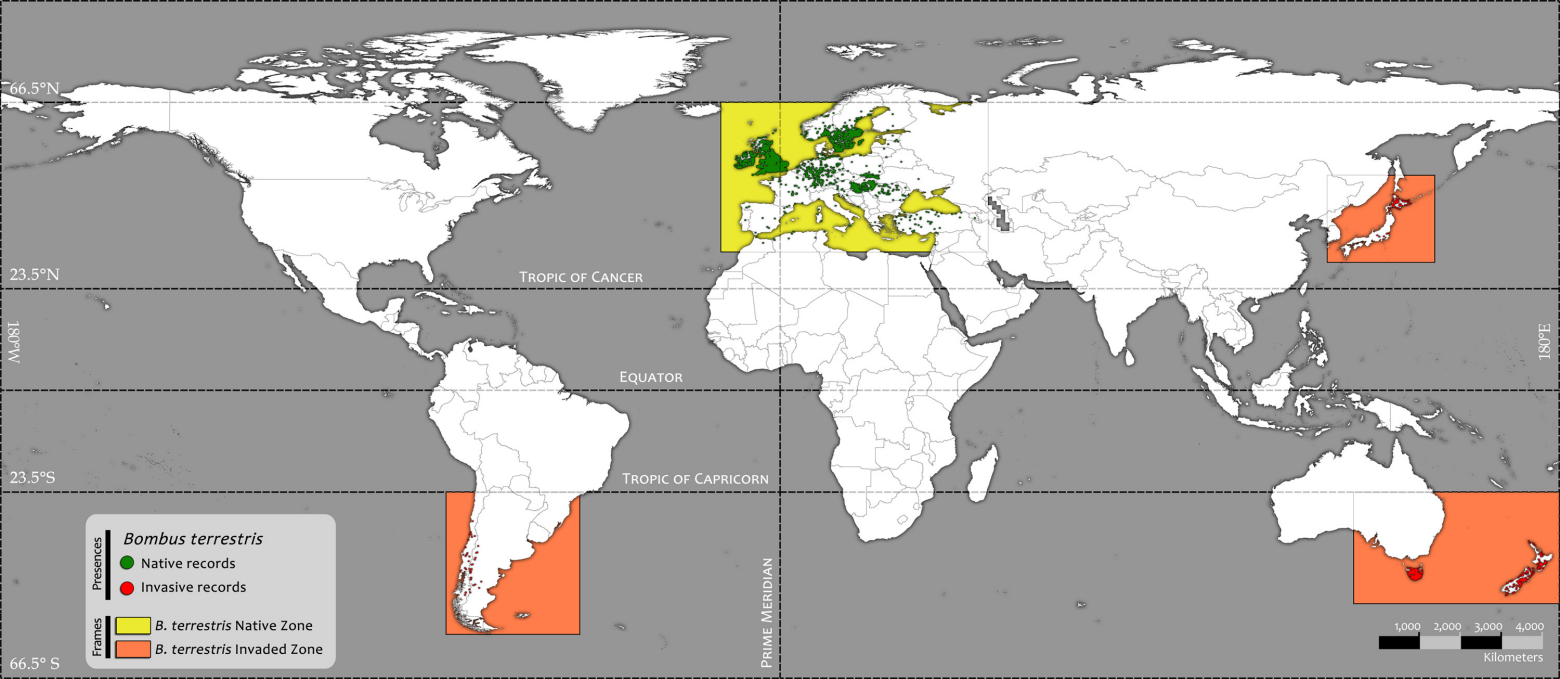
Native and invasive presence records of *Bombus terrestris* at a global scale.

At the end of our filtering process, the final dataset was composed of 4,209 native locations, 547 invaded locations (Fig 1) and 3,422 other *Bombus* spp. (i.e., *Bombus* spp. other than Bt) pseudo-absences (BOPA).

### Evaluation processes

**Fig 2:**
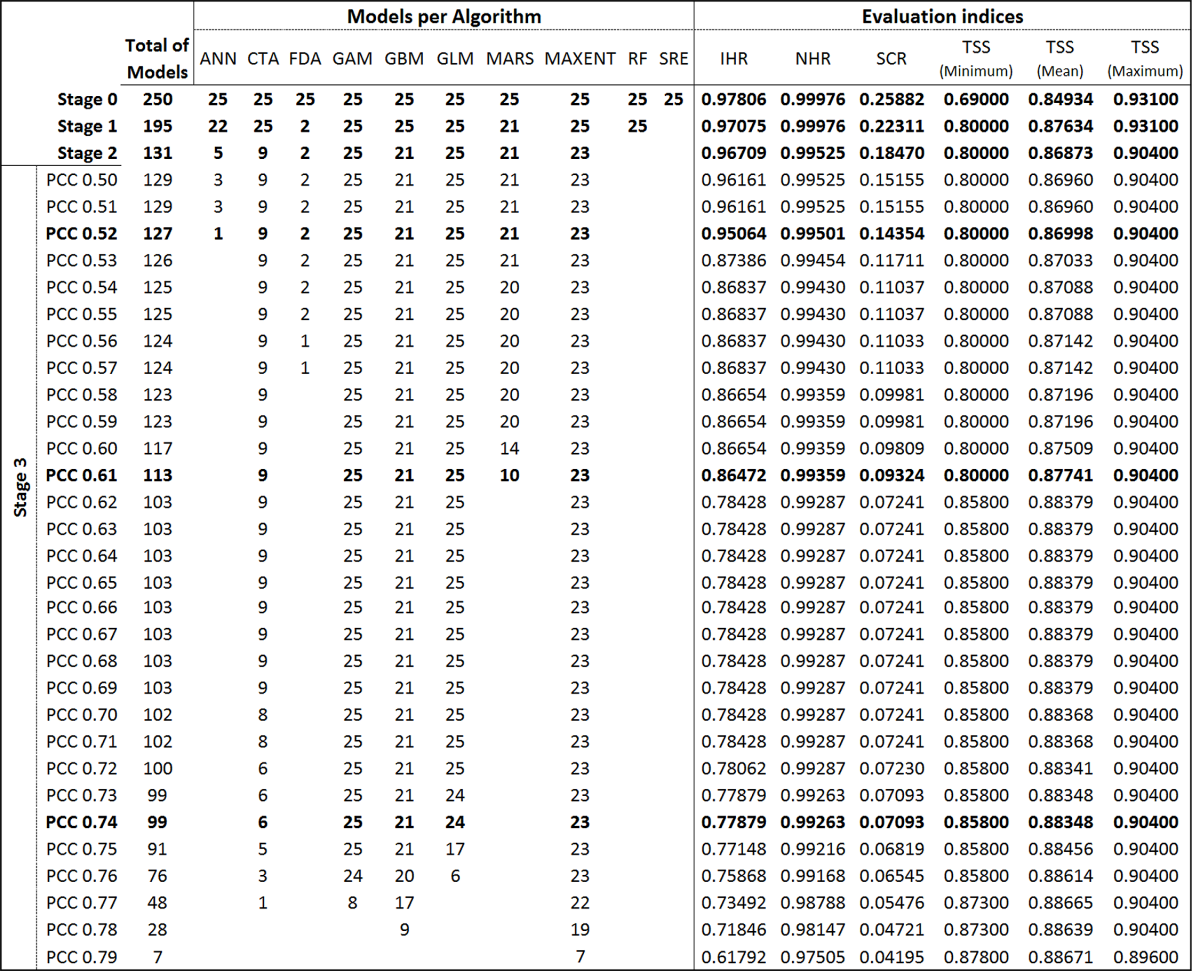
Stages (y-axis) and the respective numbers of models per algorithm, as well the respective changes in the evaluation indices.

**Fig 3:**
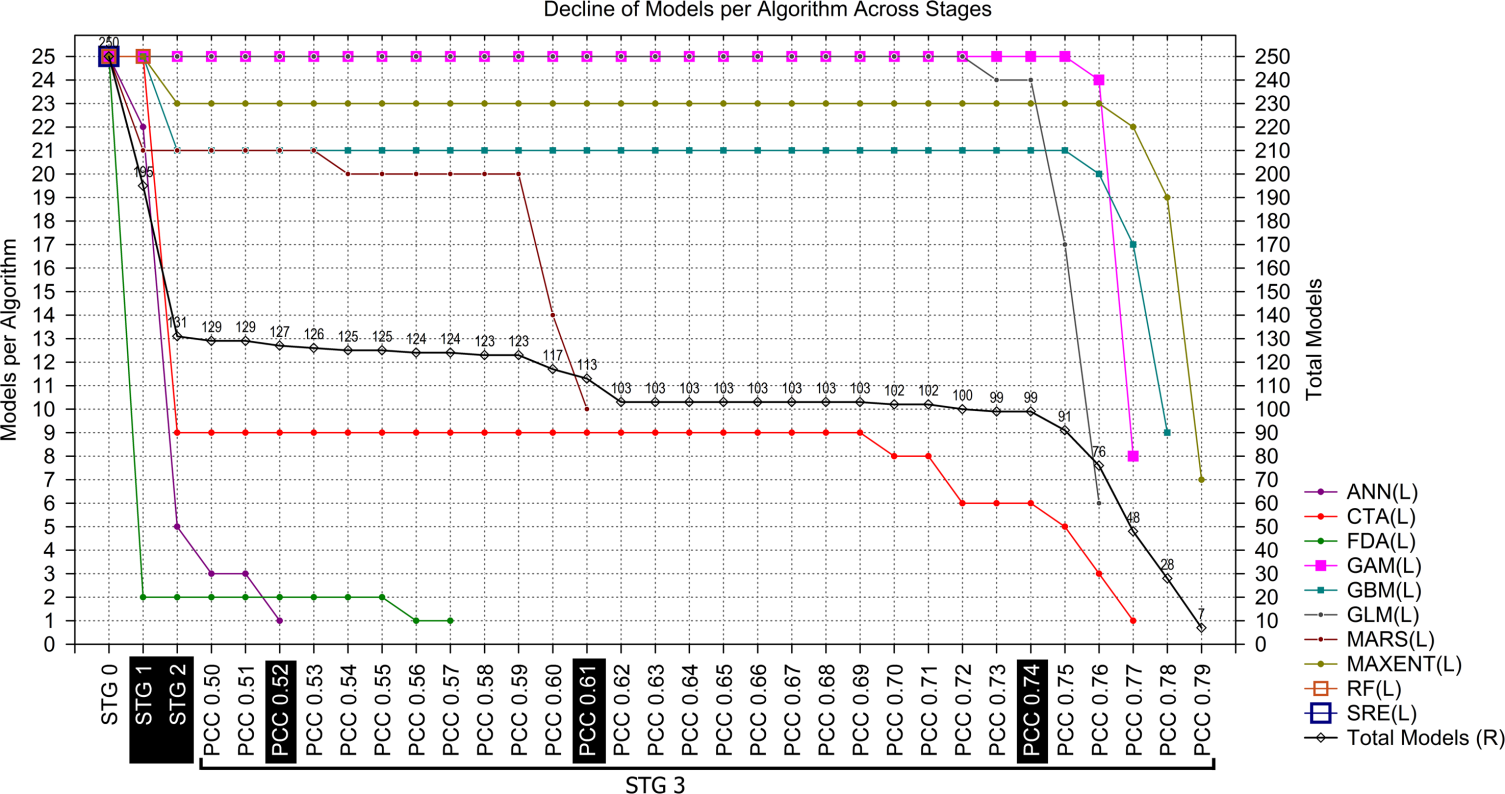
Progressive decrease of models per algorithm across stages (and across PCCs in Stage 3). Stage 0: the total number of models per algorithm from the Biomod2 output before selection. Stage 1: Models that yielded TSS ≥ 0.8. Stage 2: Models that yielded an Invasive Hit Rate (IHR) ≥ the total IHR average. Stage 3: Models that yielded a total average paired Pearson´s correlation coefficient (average of paired PCC values between itself and all others) above each predefined threshold.

The initial set of 250 models generated by 10 algorithms (Stage 0) was reduced in Stage 1 (TSS ≥ 0.8) to 195 models generated by 9 algorithms. All models from SRE were excluded, and there was a high reduction of FDA models, of which 23 were discarded (Fig 2 and Fig 3). Some small losses also occurred with ANN (loss of 3 models) and MARS (loss of 4).

In Stage 2 (Invasive Hit Rate), an extreme reduction of models occurred (loss of 64 models from Stage 1) compared to the loss in the previous stage (loss of 55 from Stage 0); thus, 131 models remained from the initial number. With the exception of FDA, GAM, GLM and MARS, all algorithms lost models: ANN lost 17; CTA, 16; GBM, 4; MAXENT, 2; and RF, all 25 models.

In Stage 3 (convergence of suitability predictions), the Pearson´s Correlation Coefficients (PCC) range had an upper bound of 0.79, as no model pair yielded values higher than this. Thus, 30 Overall Predictions Models (OPMs) were selected.

**Fig 4:**
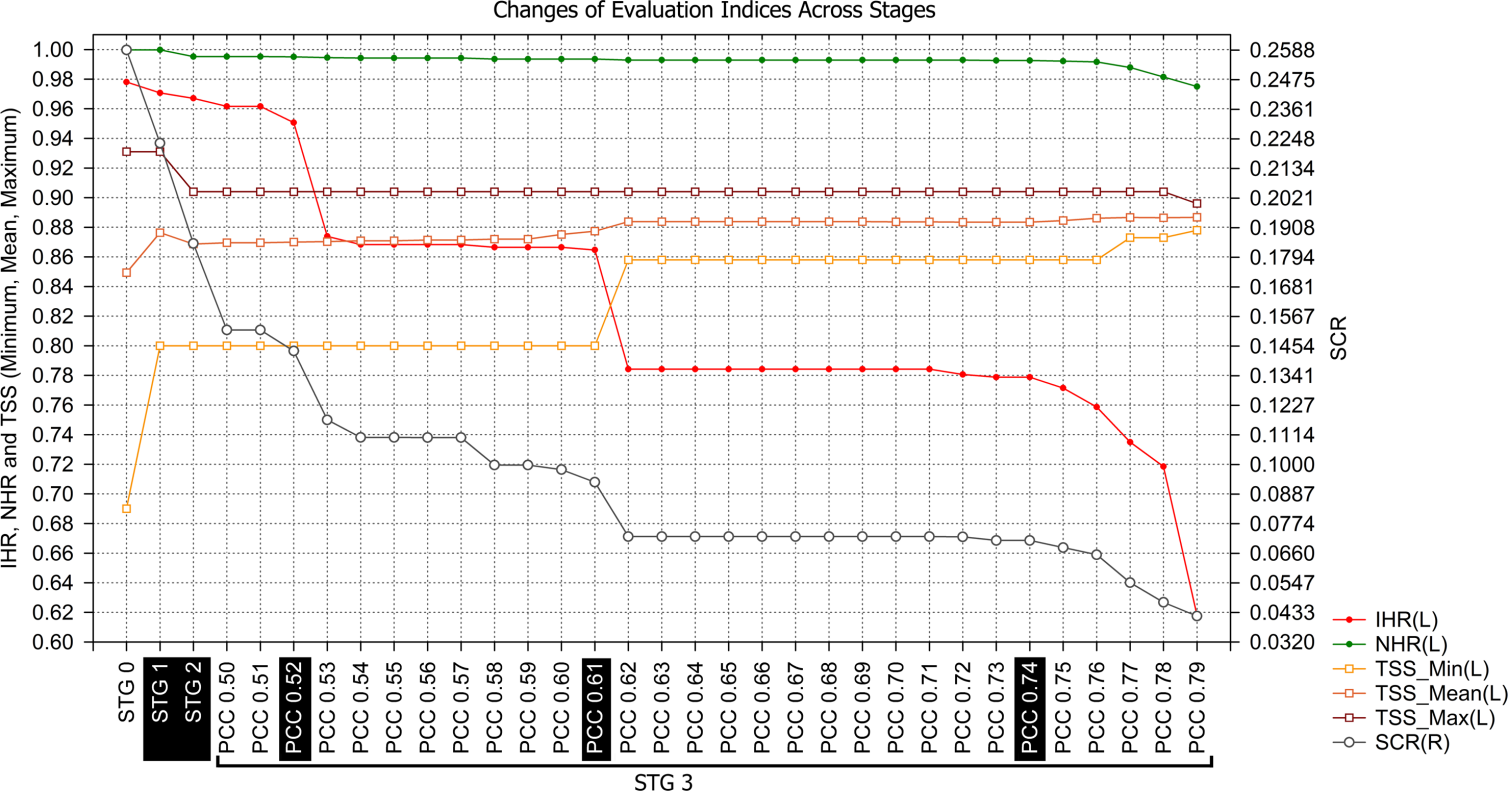
Shifts in the evaluation indices per model (IHR; NHR; SCR; minimum, mean and maximum TSS) across stages. Each evaluation index was recalculated at each step, but the TSS (minimum, maximum, mean) values were obtained at each step from the evaluation results of the Biomod2 output.

Throughout the entire evaluation range, i.e., from Stage 0 to the last PCC threshold in Stage 3, the minimum TSS increased at four points (Fig 2 and Fig 4). However, two increases were marked. The first occurred in Stage 1, when models with TSS ≥ 0.8 were excluded from the Biomod2 output models. The second occurred at the 0.62 PCC threshold (Stage 3) and was related to the marked reduction in the area of predicted suitable habitat from PCC 0.61 to 0.62 (minimum TSS from 0.800 to 0.858; Fig 2 and Fig 4) and the small SCR sequential reduction in the previous PCC thresholds, beginning beyond PCC 0.59 (Fig 4).

Across the evaluation range, the maximum TSS had one marked decrease event in Stage 2 (falling from ~0.931 in Stage 1 to ~0.904 in Stage 2; Fig 2) due to the exclusion of some high-scored models that correctly detected the native distribution of Bt (based on TSS values). However, the same excluded models only weakly detected suitability in locations of reported invasive Bt presence (based on Invasive Hit Rate values). For example, all 25 RF models yielded high TSS values and passed through Stage 1 (TSS ≥ 0.8) without model losses (Stage 0 minimum and maximum RF TSS values of 0.907 and 0.931, respectively; Fig 2). However, after Stage 2, no RF models remained (Fig 2 and Fig 3). Subsequent to this decrease, the maximum TSS remained constant over almost the entire evaluation range until the last PCC threshold (Stage 3—PCC 0.79), where a small decrease occurred. The stabilization of the maximum TSS over almost the entire PCC range is due to the permanency of a single high-TSS evaluated model since Stage 2 (MAXENT, PA 4, RUN 3; S1 Fig) that was only excluded from the set after PCC 0.78.

As expected, the mean TSS was influenced by variation in its extreme values (minimum and maximum TSS) but provided an indication of the variation in TSS central tendency of models in each set across the evaluation range. The mid-range TSS variation became evident from the absent relationship between the observed interval of mean TSS variation and the changes in extreme TSS values. For example, from Stage 2 to 0.61 (PCC, Stage3) there were small but progressive increases in the mean TSS (Fig 2 and Fig 4) that were unrelated to maximum and minimum TSS variation but instead related to the exclusion of some lower-evaluated models in the TSS mid-range. This emphasizes the overall improvement in model accuracy across the evaluation range within the most central range of TSS values.

Decreases in the Native Hit Rate (NHR) were relatively minimal across the entire evaluation range (Fig 4 and Fig 2), the total decrease reaching only approximately 2.5%. This indicates that, from the initial number of 4,209 Bt native presence records used, 4,208 intersect suitable areas predicted by at least one model in Stage 0 (S2 Fig). From this value (Stage 0), only 104 hits were lost before the last PCC threshold (PCC 0.79, Stage 3; S2 Fig). Small NHR changes were observed at many points across the evaluation range (Fig 2), but only four were relatively marked: one in Stage 2 and three in the final PCCs of Stage 3 (PCCs 0.77, 0.78 and 0.79)(Fig 4 and Fig 2).

The Suitable Cells Ratio (SCR) exhibited the highest variability among all evaluation indices except in the range from PCC 0.62 to 0.72, where we observed a pattern of SCR stability (Fig 2). The SCR scale (Fig 4 and Fig 2) does not provide a clear representation of the spatial scale contraction; for example, from Stage 1 to 2, a decrease of approximately 7.5 million km^2^ of suitable area was estimated (grid cell size at equator)(S2 Fig).

We observed positive and negative influences in the evaluation indices related to the reduction of OPMs suitable areas (SCR) across the PCC range (Stage 3)(S3 Fig). The SCR variation exhibited a strong linear relationship (Pearson´s r) with IHR (r:~ +0.965; p = 6.9E-18) as well as strong, but negative, relationships with the mean TSS (r:~ -0.929; p = 1.1E-13) and the minimum TSS (r:~-0.884; p = 9.6E-11). The SCR exhibited a weak relationship with NHR (r: ~ +0.621; p = 0.00024) and a non-significant relationship with maximum TSS (r: ~ +0.299; p = 0.1).

**Fig 5:**
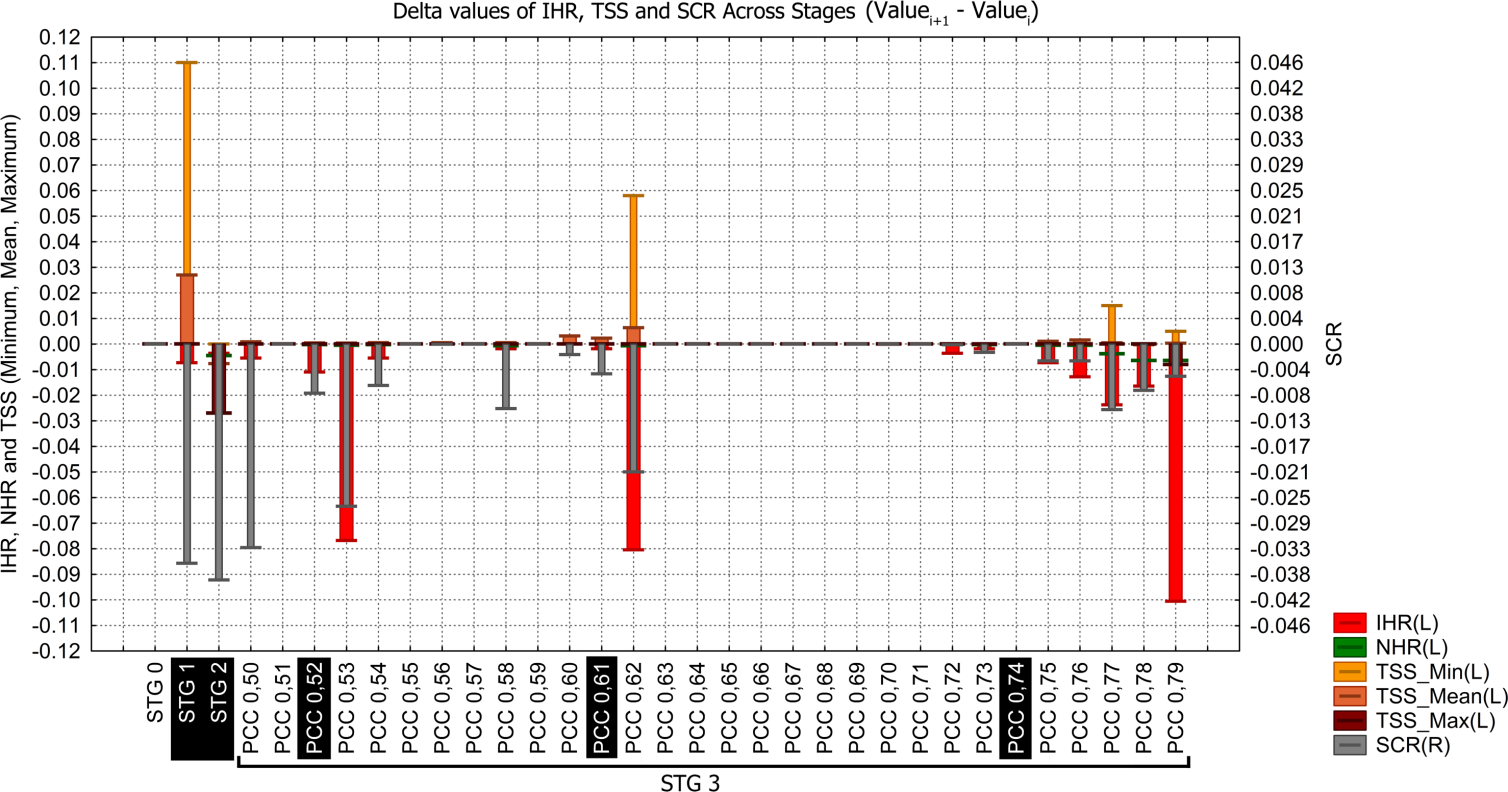
Difference between the current value minus the preceding one (delta value) of each evaluation index across stages. This plot facilitated the identification of three marked changes across the PCC range (PCC: 0.53, 0.62, 0.79) and the sequence of changes from PCC 0.75 to 0.79 used to select the OPMs (PCC: 0.52; 0.61; 0.74) that built the final ensemble model.

Invasive Hit Rate (IHR) variation and its relationships with other indices are depicted in Fig 5, where each evaluation index value was subtracted from the previous one across the evaluation range (Value_i+1_—Value_i_).

Some decreases in IHR occurred across the evaluation range, mainly related to SCR decreases, but three extreme decrease events were apparent at PCC 0.53, 0.62 and 0.79. The latter PCC (0.79) is preceded by a sequence of decreases starting at PCC 0.75. We detected three PCC thresholds that represent the main changes that occurred across the Stage 3 evaluation range (Figs 4 and 5; Fig 2 and S2 Fig). The first one occurred at PCC 0.53, with a pronounced decrease in IHR relative to the previous PCC threshold, indicating a reduction of 42 invasive presences hitting suitable habitats (S2 Fig). Prior to this point, only 15 invasive hits were lost (Stage 0 to PCC 0.52). This event was also associated with a strong decrease in the Suitable Cells Ratio (SCR), an approximately 18.4% reduction in the global suitable area predicted from the previous PCC (SCR from ~0.14 to ~0.11; Fig 2). Based on this pronounced shift event in the evaluation indices, we chose the OPM positioned immediately preceding it to compose the final ensemble model (PCC 0.52). The second threshold occurred at PCC 0.62 (Figs 4 and 5), with an even more pronounced IHR decrease, resulting in a loss of 44 invasive hits from the previous PCC, which was also followed by a greater suitable area (SCR) reduction than the last event, approximately 22.3% from the previous PCC threshold (PCC 0.61; Fig 2). Thus, we also chose the PCC 0.61 OPM to compose the final ensemble model. In the third case, we choose the PCC 0.74 OPM, which is positioned in the evaluation range just before the beginning of successive events of Invasive and Native Hit Rates (IHR and NHR) decreases, initiated at PCC 0.75 (Fig 2; Figs 4 and 5). For each respective selected threshold in Stage 3 (PCC 0.52, 0.61 and 0.74), the OPMs were composed by 127 models generated by 8 algorithms; 113 by 6 algorithms; and 99 by 5 algorithms (Models per Algorithms in Fig 2 and Fig 3).

From the ten algorithms used in the modeling procedure, only four contributed at least 20 models (≥80% of total models per algorithm) to all selected models (OPMs): GAM, GLM, MAXENT and GBM (Fig 2). Considering also the algorithms that contributed more than 20 models for at least one selected OPM, we added MARS to this list, with 21 models only at PCC 0.52 (Fig 2 and Fig 3). Thus, we considered these five algorithms to be more reliable for extrapolating habitat suitability predictions over more distant areas from the native distribution range of Bt, particularly the first four: GAM, GLM, MAXENT and GBM.

### From OPMs to Global Susceptibility Map to *Bombus terrestris* Invasion

**Fig 6:**
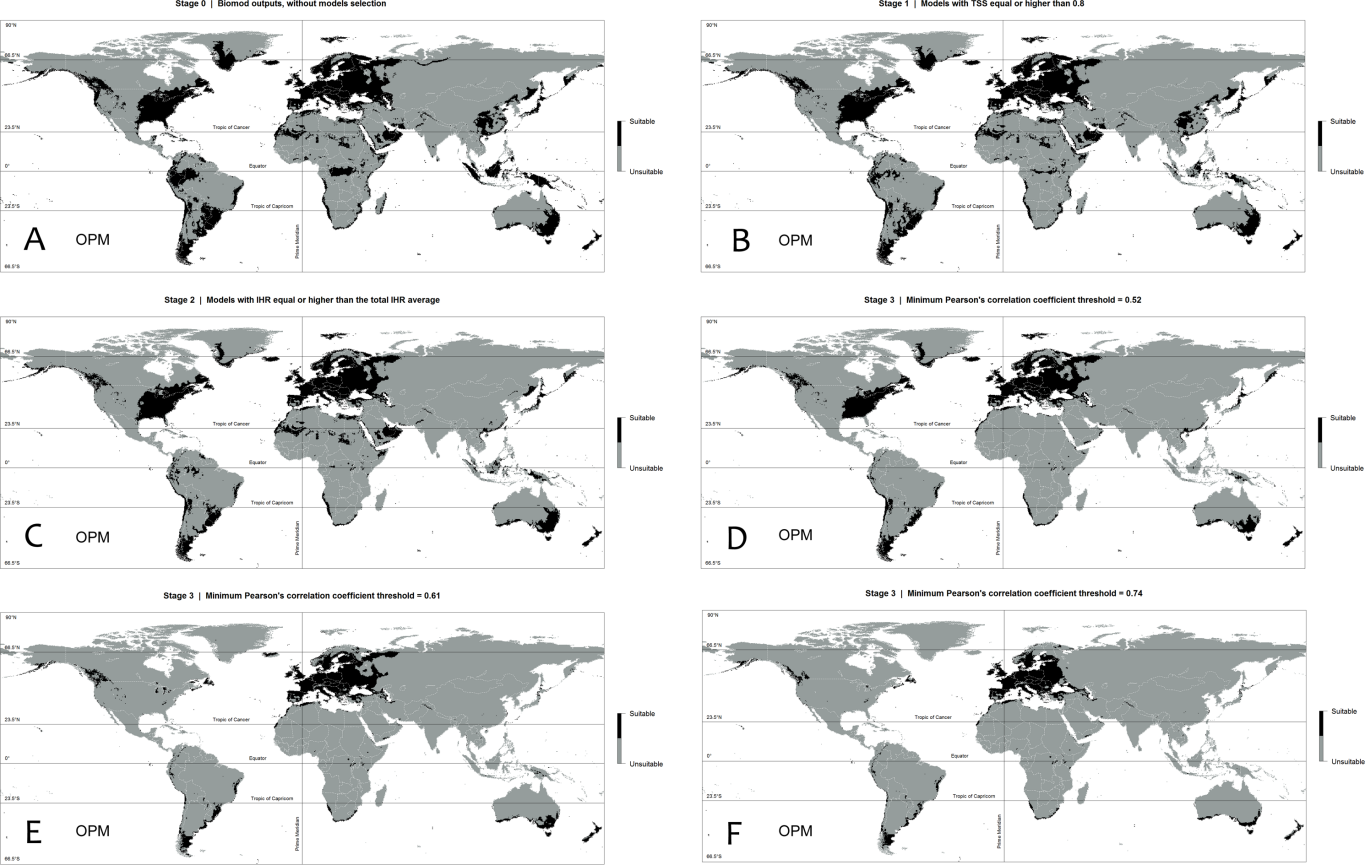
Overall Prediction Ensemble Models (OPMs) of Stages 0, 1, 2, and Stage 3 PCCs 0.52, 0.61, and 0.74. The OPM considers all suitability predictions of every model from each selected set per Stage.

**Fig 7:**
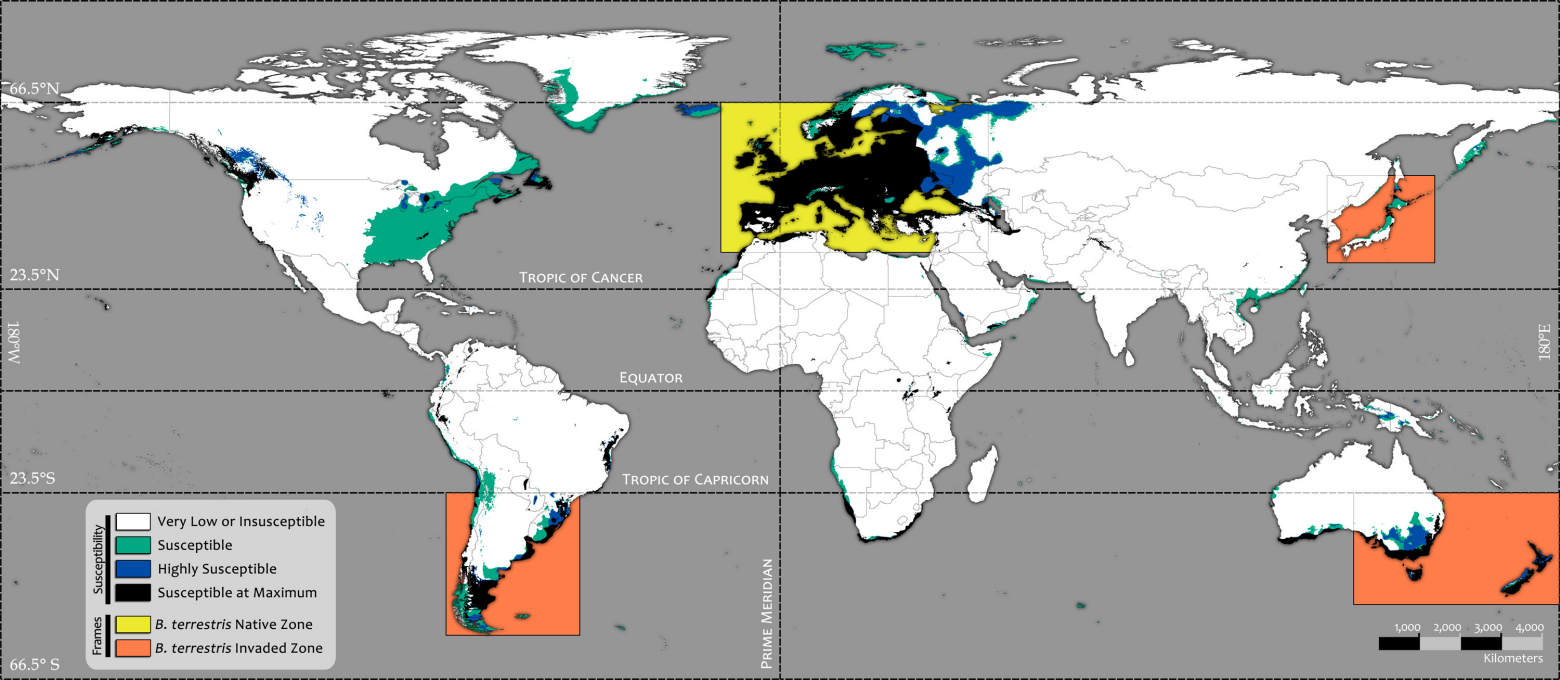
Global Susceptibility Map to *Bombus terrestris* Invasion.

**Fig 8:**
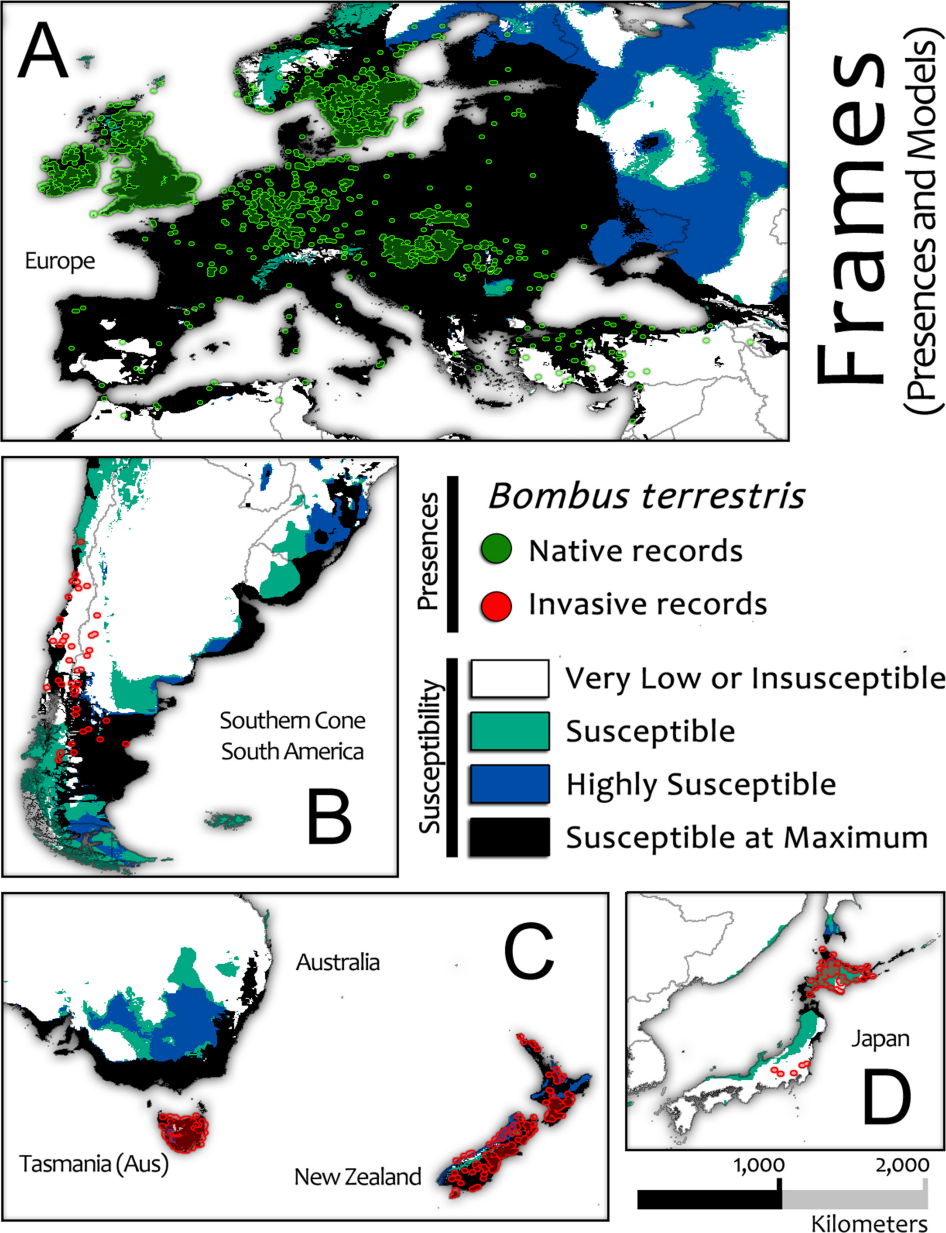
Frames extracted from the Global Susceptibility Map to *Bombus terrestris* Invasion with both invasive and native presence records plotted.

We used the three selected OPMs to ensemble our main model (D, E and F in Fig 6), which was projected as a Global Susceptibility Map to *Bombus terrestris* Invasion (global view in Fig 7 and framed in the spatial range of native and invasive presences in Fig 8). The Agreement Level Ensemble Models (ALMs), which were used to build the OPMs, were also projected (see S4 Fig).

As each OPM contains the susceptible area predicted by the set of models of each subsequent OPM, the OPM from PCC 0.52 models predicted the largest susceptible area to Bt invasion on a worldwide scale (Fig 6 D). This covered approximately 28 million km^2^ (cell size estimated at the Equator; S2 Fig), and its susceptible area intersects 4,188 native (approximately 99.5%) and 520 invasive presence records (approximately 95%) of the initial total (Fig 2 and S2 Fig). However, the susceptible area identified exclusively by this OPM (class value = 1 after the OPMs sum; green areas in Fig 7) is more spatially restricted and marginally distributed, covering approximately 9.9 million km^2^ and intersecting only 6 native (approximately 0.14%) and 47 invasive presence records (approximately 8.6%) of the initial total. We classified this area as **Susceptible** for the Global Susceptibility Map to Bt Invasion (Fig 7).

The second largest susceptible area to Bt invasion was provided by the OPM PCC 0.62 (Fig 6 E), covering approximately 18 million km^2^ and intersecting 4,182 native (approximately 99.3%) and 473 invasive presence records (approximately 86.4%) of the initial total (Fig 2 and S2 Fig). Additionally, in this case, the exclusive susceptible area identified by this OPM plus the susceptible area from the previous one (class value = 2; blue areas in Fig 7) are even more spatially restricted, covering approximately 4.4 million km^2^ and intersecting 4 native (approximately 0.09%) and 47 invasive presence records (approximately 8.6%) of the initial total. This area was classified as **Highly Susceptible** (Fig 7).

The susceptible area shared by all OPMs together, represented by the OPM PCC 0.74 prediction (class value = 3 after the OPMs sum; Fig 6 F; black areas in Fig 7), covers approximately 13.9 million km^2^ and contains the majority of the Bt presence records. This area exclusively intersects 4,178 native (approximately 99.2%) and 426 invasive Bt records (approximately 77.9%) and was classified as **Susceptible at Maximum** (Fig 7).

The largest shared area among all selected OPMs, where all models agreed with *no suitability* and consequently *no or very low susceptibility* to Bt invasion was classified as **Very Low Susceptibility or Insusceptible** to Bt invasion (white areas in Fig 7).

## Discussion

Our methodological approach based in the three-stage selection criteria reduced the presence of statistical artifacts and incoherent predictions, retaining models with high predictive accuracy and high extrapolative capacity, i.e., the best models for delineating the global map of areas susceptible to Bt invasion. Moreover, it also showed that four algorithms (GAM, GLM, MAXENT and GBM) provided the best results, yielding models with high predictive convergence among them and high predictive and extrapolative capacity.

### Overall performance of the methodological framework

The three-stage procedure resulted in an overall improvement of TSS values, with minor decreases events in the maximum and mean TSS, as well some small losses in the native and invasive hit rates. However, even the lowest hit rate exhibited by the most conservative level of susceptibility can be considered a good score. In fact, the area classified as the maximum level of susceptibility contains the largest number of invasive and native Bt presence records. Moreover, it can be considered the most reliable representation of global susceptibility to Bt invasion, due to the highest prediction convergence among models and algorithms.

Stage 1 mainly excluded the models of lowest quality in terms of suitability prediction near the native Bt distribution. However, TSS did not detect models that were unable to extrapolate predictions to more distant areas nor did it detect some models with statistical artifacts and incoherent predictions (see S1 Text). Thus, Stage 2 was able to detect and filter out the models with low capacity to extrapolate suitability. However, we observed a small decrease in the Invasive Hit Rate (IHR) when compared with the previous stage, this being an undesirable but interesting result. This difference arises from the exclusion of some individual models in the Stage 2 due to their below-average IHR that nonetheless correctly predicted some specific fragments of suitable area covering invasive presence records. However, the selected models, i.e., those with scores greater than the total average IHR, were unable to predict suitability in those same specific fragments. Despite predicting some specific suitable areas that others did not, these models were correctly excluded at this stage due to their low overall capacity to extrapolate suitable areas. The main advantage of Stage 3 was the ability to detect and exclude models with highly divergent predictions when considering the majority of model predictions. These divergent predictions were mostly related to statistical artifacts and to under- and over-fitting. Therefore, the progressive reduction in these undesired statistical effects was directly related to the increase in the Pearson´s Correlation Coefficient (PCC) thresholds. However, a sequence of decreasing accuracy (IHR and NHR) is initiated above PCC 0.75, related to the extreme suitable area contraction (SCR). This suggests that in our case, PCC 0.74 is the last acceptable (accurate) threshold in the range.

Overall, our framework aggregated different mathematical logics (from ten algorithms) and variable input data (PAs and Bt presence partitioning) that yielded convergent results in the final sets of selected models. We consider the susceptibility levels of invasion obtained here to be more robust and accurate than the categorization (into classes) of continuous probabilistic approaches, even though we did not conduct a formal comparison. Additionally, we verify the performance of each particular algorithm across the evaluation criteria; this information can be consulted in S1 Text.

### Global susceptibility to *Bombus terrestris* invasion

Areas susceptible to Bt invasion are almost entirely limited to the north and south temperate climate zones, suggesting the possible restriction of Bt invasion to tropical environments. If we consider exclusively the range of the native Bt zone, it is apparent that almost all suitable area is restricted to the Europe continent and western Russia, and the Susceptible at Maximum level covers almost exactly the entire native Bt distribution, emphasizing that Bt is predominantly a temperate species.

Large suitable areas were predicted in the easternmost region of the area of native Bt records used in the modeling, mainly from the northwest (Murmansk) to the southwest (Dagestan) of the Russian Federation. There was also a big suitable area covering the region of Moscow city. Nevertheless, we found no presence records there, despite the reporting of Russia as a native environment for Bt [40].

In the southeastern region, suitable areas were predicted surrounding the continental seas of Azov, Caspian and Marmara; the Black Sea; and areas in Georgia, Azerbaijan, Syria and Lebanon. We found no reports of native Bt presence in these countries. However, these areas are close to Turkey, a country with many records and reports of native Bt presence [40, 96, 97].

In the southern area of native Bt records, specifically south of the Mediterranean Sea, most of the relatively small suitable areas were identified in northern Algeria, northwest Morocco and the Western Sahara, along with small areas in Tunisia, the Gaza Strip, Libya, Saudi Arabia, Jordan and Egypt. Among these countries, Morocco, Tunisia, Saudi Arabia and Jordan are reported as non-native areas for Bt, with evidence of Bt invasion [40]. Libya and Egypt have been reported as countries without Bt [64].

Only a very low susceptibility was detected for Israel, a country that uses Bt colonies for greenhouse pollination and where Bt is reported as invasive [40, 42, 49, 64, 98, 99]. Considering that the reported invasion of Bt in Israel is in the north of the country, mainly in the region of forest fires at Mt. Carmel [64], the resolution of variables may have been insufficient for detecting the particular topoclimatical conditions of this upland zone.

Reported occurrences of invasive Bt in South America were predominantly distributed in southern Chile and southwestern Argentina, but we detected large susceptible areas in eastern and southern Argentina, in central and eastern Uruguay, and in southeastern Brazil. These susceptible areas are almost entirely connected to invaded regions. Additionally, a large coastal corridor classified as *Susceptible at Maximum* connects the invaded regions to Uruguay and Brazil.

The first report of invasive Bt presence in Argentina [62] suggested that this species reached the zone of San Carlos of Bariloche (Argentina), crossing the Andean Mountains via low-altitude pathways from Chile, where Bt was first introduced in South America. Recently, it was reported that Bt expanded rapidly (200 Km/year) and massively their range in the south of this continent; remarkably, this species spread it invasion from the western–near the Pacific coast of Chile—to the easternmost of the continent—reaching the Atlantic coast of Argentina [41]. This scenario demonstrates the well adaptation to the regional environment and the high dispersal capacity of Bt, which increases the probability of invasive expansion over these susceptible corridors detected. Furthermore, it is likely that colonies (or inseminated queens) could be carried and released into these susceptible areas, accidentally or deliberately, by humans.

There is some evidence of the spontaneous spread of Bt in Uruguay after introduction [40]; however, we found no more information about sightings of Bt specimens or colonies in this country. Recently (2013), a large survey conducted during the spring and bordering the frontier between Brazil and Uruguay was made by the author (ALA) and collaborators, aiming to find invasive Bt in the wild. We found a large number of native bee species (including other *Bombus* spp.), but we did not find any Bt specimens. Until now, there is no strong evidence of Bt presence in either Uruguay or Brazil, but there is a strong possibility that Bt could use this susceptible pathway to reach both countries, increasing its invasive distribution from Chile and Argentina.

Bt threat was also detected by our models in the temperate zone of southwestern Oceania; notably, a large susceptible area was identified in Australia, where there is no reported invasion. Although Bt invasion was reported in the islands of Tasmania (AUS), with approximately 200 km of oceanic barrier separating them from Australia, and Bt invasion reported in both main islands of New Zealand, with approximately 1700 km of ocean between them and Australia, the global model showed a large, connected susceptible area in Australia. Assuming trade and transportation among these islands, the probability of Bt invasion into Australia could be considered high, as any inseminated queen hitchhiking via a commercial ship could start an invasion in this country.

This possibility has been a concern for some time inside academic and governmental circles [99–101]. This concern could be aggravated due to planned Bt importation by private agricultural sectors for greenhouse pollination [40, 102] despite opposing initiatives (e.g., [42]). Regardless of whether Bt has yet to occur in Australia (we found no scientific reports of such), we strongly recommend monitoring and/or surveying in the susceptible area, particularly in the area of *Susceptible at Maximum Level*.

The susceptible areas detected in New Zealand and Tasmania precisely covered all reported invaded locations [13, 40, 42, 49, 51, 58, 99, 103, 104]. Recall that we did not include these invasive presence records in the modeling procedure; thus, in both countries, the predictions of susceptible areas can be considered very accurate. The susceptible areas in these islands cover almost all land except for the mountain ranges of highest altitude.

In Japan, the susceptible areas coincided with Bt invasions already reported on Hokkaido Island [15, 105, 106], mainly in the north and northeast. However, the model failed to detect the reported invasive presence on Honshu Island.

Companies that commercially produce Bt colonies for pollination in South Korea have been reported [45, 49], and there is evidence of invasive spread [40]. However, the model detected low susceptibility to Bt invasion in this area.

A large susceptible area without reports of invasive Bt presence was detected in eastern Canada and the United States. This area has some environmental similarities with the native range of Bt, but almost all of the susceptible area detected was through a single model (from ANN); thus, we suggest that the classification for this area as suitable be considered with caution. However, we cannot disregard this prediction because other models in the selected set also identified some susceptible areas in these countries.

A long strip of susceptibility in the southeast of China and a wide contour band of Susceptible at Maximum in South Africa were detected; both countries are reported as threatened by Bt invasion [40, 42]. Bt has already been introduced into South Africa, but there is no evidence of invasive spread [40]. Nevertheless, based on the model, spread is likely to occur, eventually reaching southern Namibia. Companies developing industrial pollination have been reported as commercializing Bt colonies in China [49] and according to our models, it is likely that invasive Bt will spread through the susceptible area in the temperate zone, including Taiwan.

Many other susceptible areas were detected in countries and regions of temperate climate, but we found no information on commercial Bt colonies or sightings of Bt individuals for these areas. Examples include a narrow strip of high susceptibility in the west of the Himalaya Mountain Range covered by the Kashmir Region in India and northeastern Pakistan; a large susceptible area covering Iceland, Greenland and Svalbard (Norway); small areas in Iran and in the easternmost zone of Russia (Kamchatka Krai) and various small islands. In contrast, in tropical zones, the potential for Bt invasion can be considered very low. However, we cannot discard the possibility for some areas, especially if Bt colonies are introduced by humans. For example, in the highlands region of Lake Eduard (Democratic Republic of Congo), where the temperature is mild and the precipitation is high, the models detected a maximum level of susceptibility. This also applies to South American areas and the tropical highlands of the Andes.

## Conclusions

The framework developed here presents new insights into multi-modeling methodological approaches of habitat suitability, mainly suggesting new criteria for pseudo-absences generation and model evaluation and selection to build a unique ensemble model with improved prediction accuracy. This approach can be easily implemented in existing robust platforms of Habitat Suitability Modeling.

The global map of susceptible areas can aid the design of more effective action plans for monitoring Bt invasion. It is important to consider public campaigns involving local people, which could contribute to a broader campaign for monitoring invasion over a large area. For example, Australia, Brazil and Uruguay could use the map to develop monitoring and mitigation actions that prioritize the border regions of areas already invaded.

The modeling component of the framework is not limited to the use of topoclimaticvariables; others bionomics factors can be added if necessary. However, the method is not fitted to analyze rapid evolutionary process that species can potentially exhibit in the new invaded environments, which can be relevant to estimate the invasive process at medium to long term.

Finally, the framework proposed here can readily be adapted to other invasive species for predicting and monitoring spread. These actions could contribute to protect biodiversity and, in the case of *Bombus terrestris*, helping to reduce and avoid further threats to native bees, safeguarding their indispensable services for ecosystems and human food security.

## Supporting Information

S1 Fig Biomod2: TSS evaluation output. TSS values per algorithm (250 models = 5 pseudo-absences dataset x 5 training and test partitioning of the native presence records x 10 different algorithms).
(TIF)

S2 Fig Presences hits and suitable habitat area. Stages (y-axis) and the number of invasive and native presence records hitting suitable areas per each respective Stage OPM generated, as well as the raw number of suitable cells per model, the total suitable area (km2) and the difference in suitable area predicted from the current model minus the previous one (delta values).
(TIF)

S3 Fig Pearson correlation between paired evaluation indices. (TIF)

S4 Fig Agreement Level Models (ALM). (TIF)

S1 File Data providers and bibliographical sources surveyed for *Bombus terrestris* native and invasive occurrences. (DOCX)

S2 File Biomod2: Algorithms Parameters. (DOCX)

S1 Text Algorithms performance. (DOCX)
