# Supplementary figures and images for: Worldwide Alien Invasion: A Methodological Approach to Forecast the Potential Spread of a Highly Invasive Pollinator
Source: PLoS One. 2016 Feb 16;11(2):e0148295. doi: 10.1371/journal.pone.0148295 (PMC4755775; doi:10.1371/journal.pone.0148295)

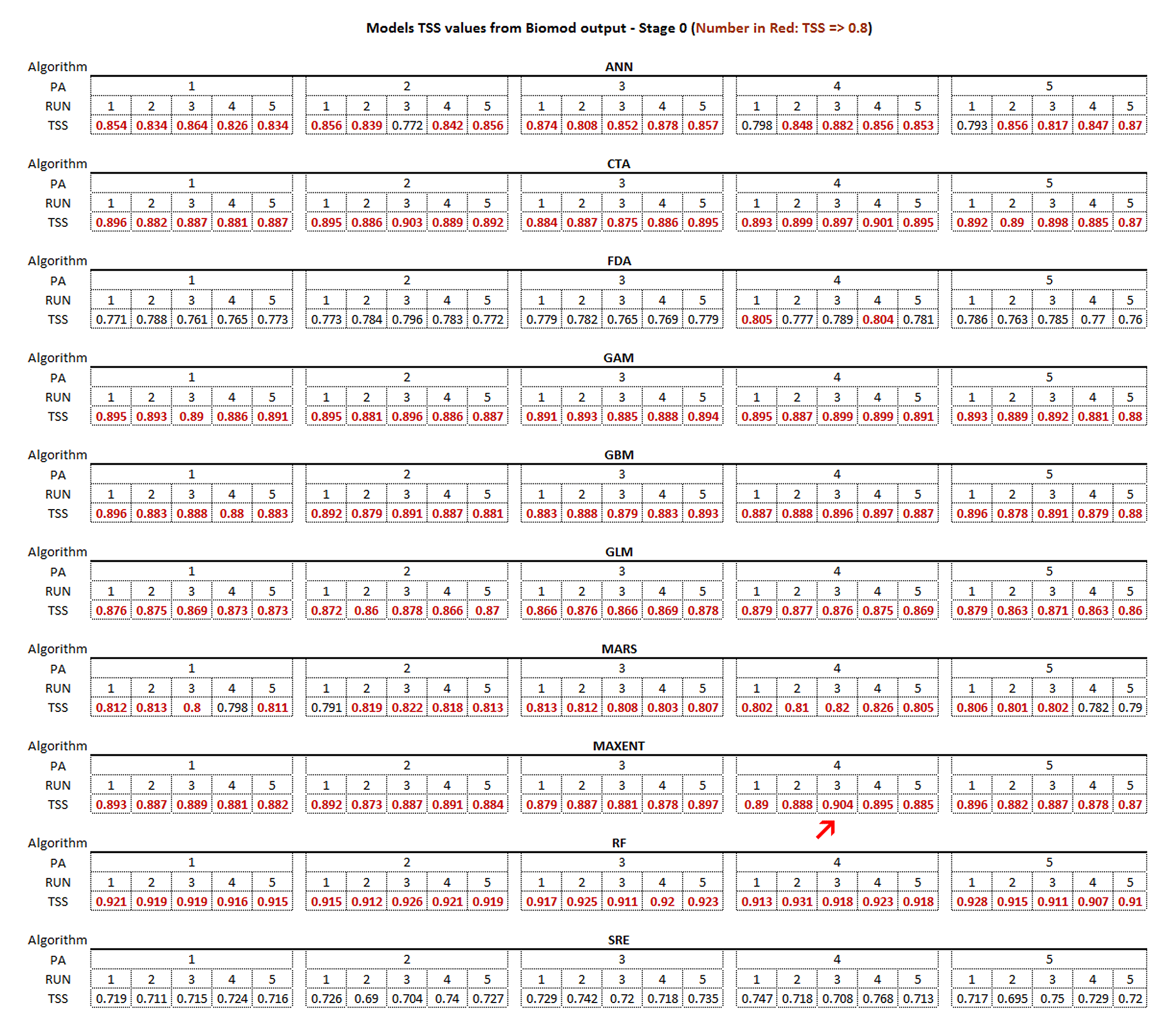

Supplement: S1 Fig — TSS values per algorithm (250 models = 5 pseudo-absences dataset x 5 training and test partitioning of the native presence records x 10 different algorithms). (TIF) [file pone.0148295.s001.tif]

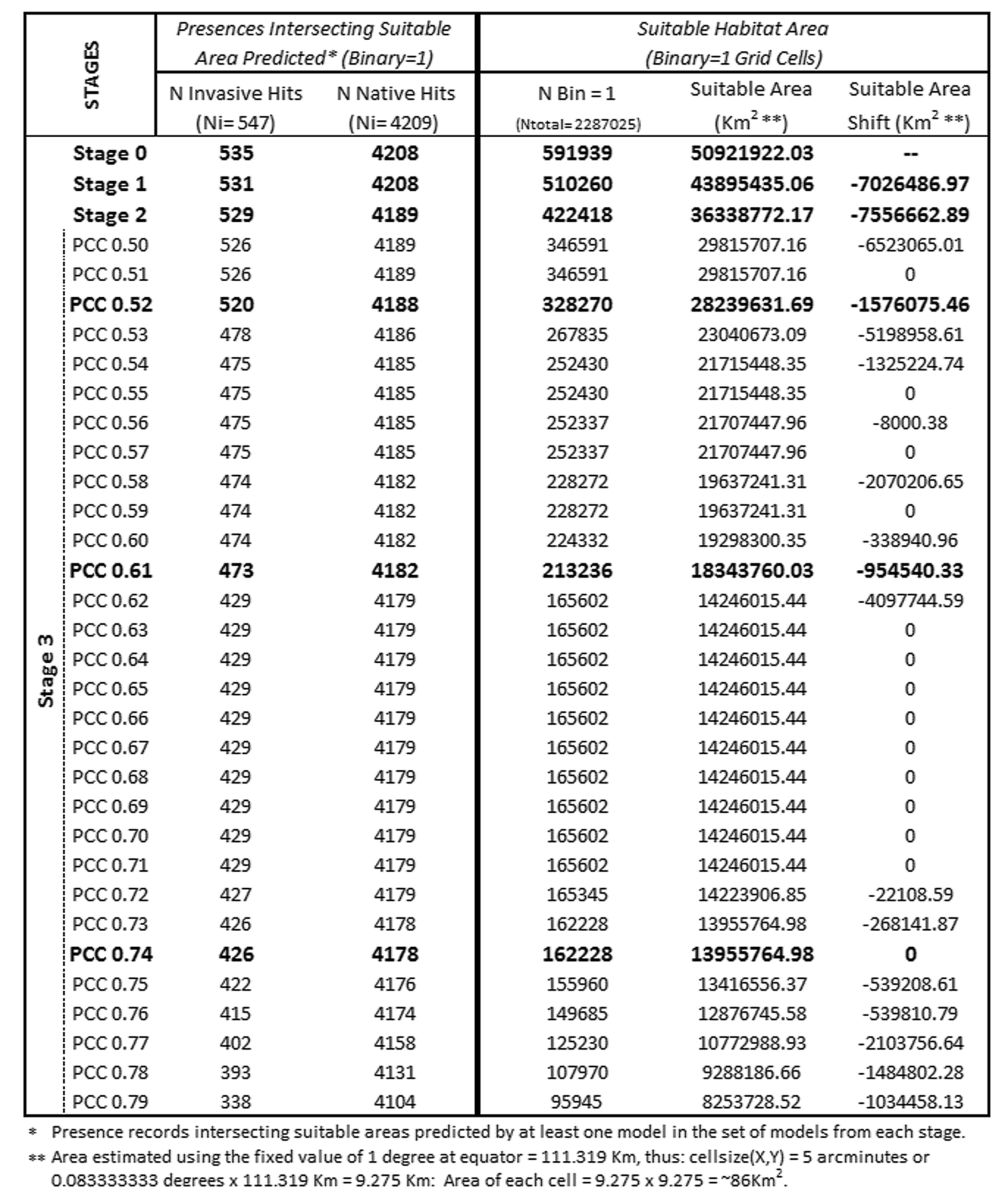

Supplement: S2 Fig — Stages (y-axis) and the number of invasive and native presence records hitting suitable areas per each respective Stage OPM generated, as well as the raw number of suitable cells per model, the total suitable area (km2) and the difference in suitable area predicted from the current model minus the previous one (delta values). (TIF) [file pone.0148295.s002.tif]

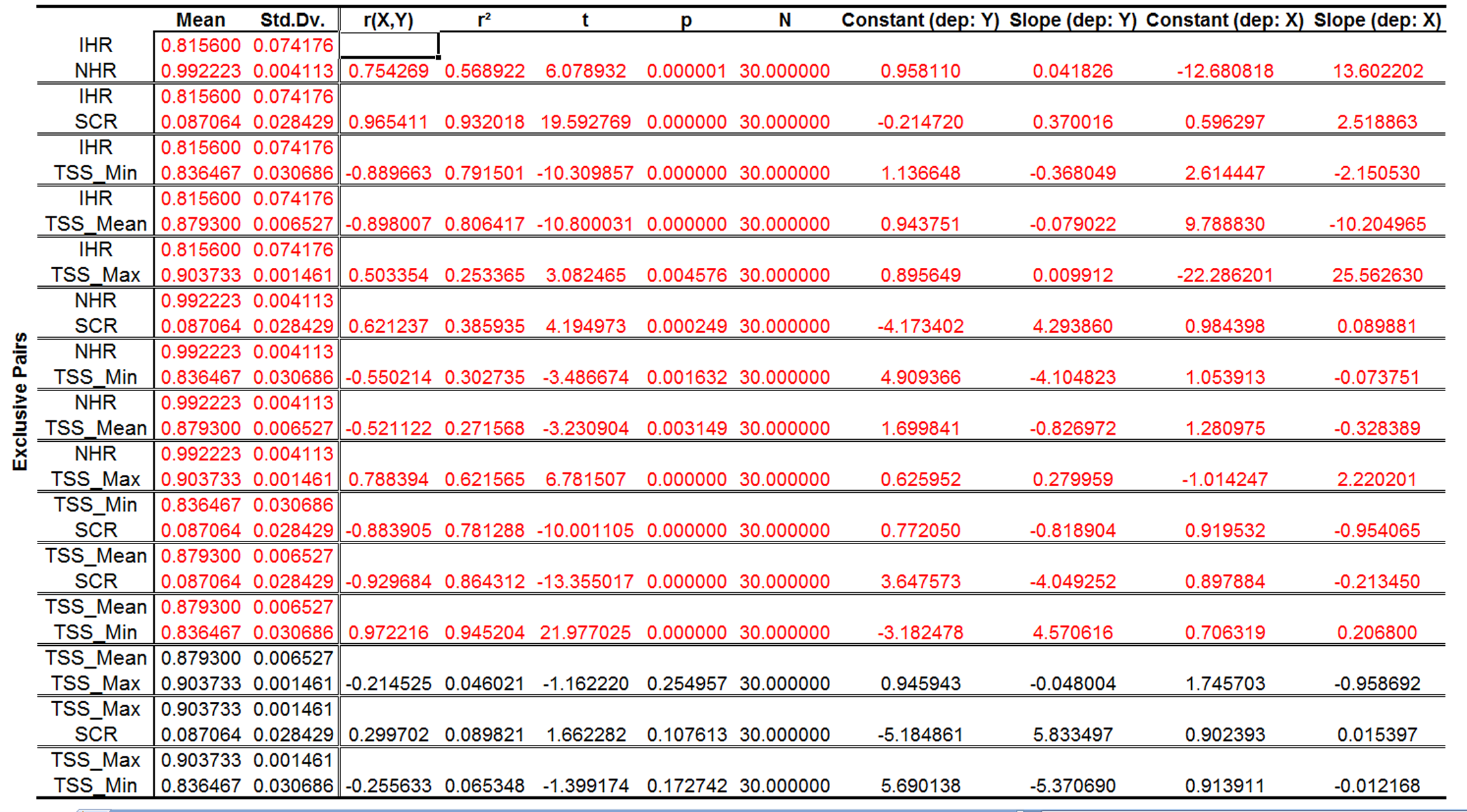

Supplement: S3 Fig — (TIF) [file pone.0148295.s003.tif]

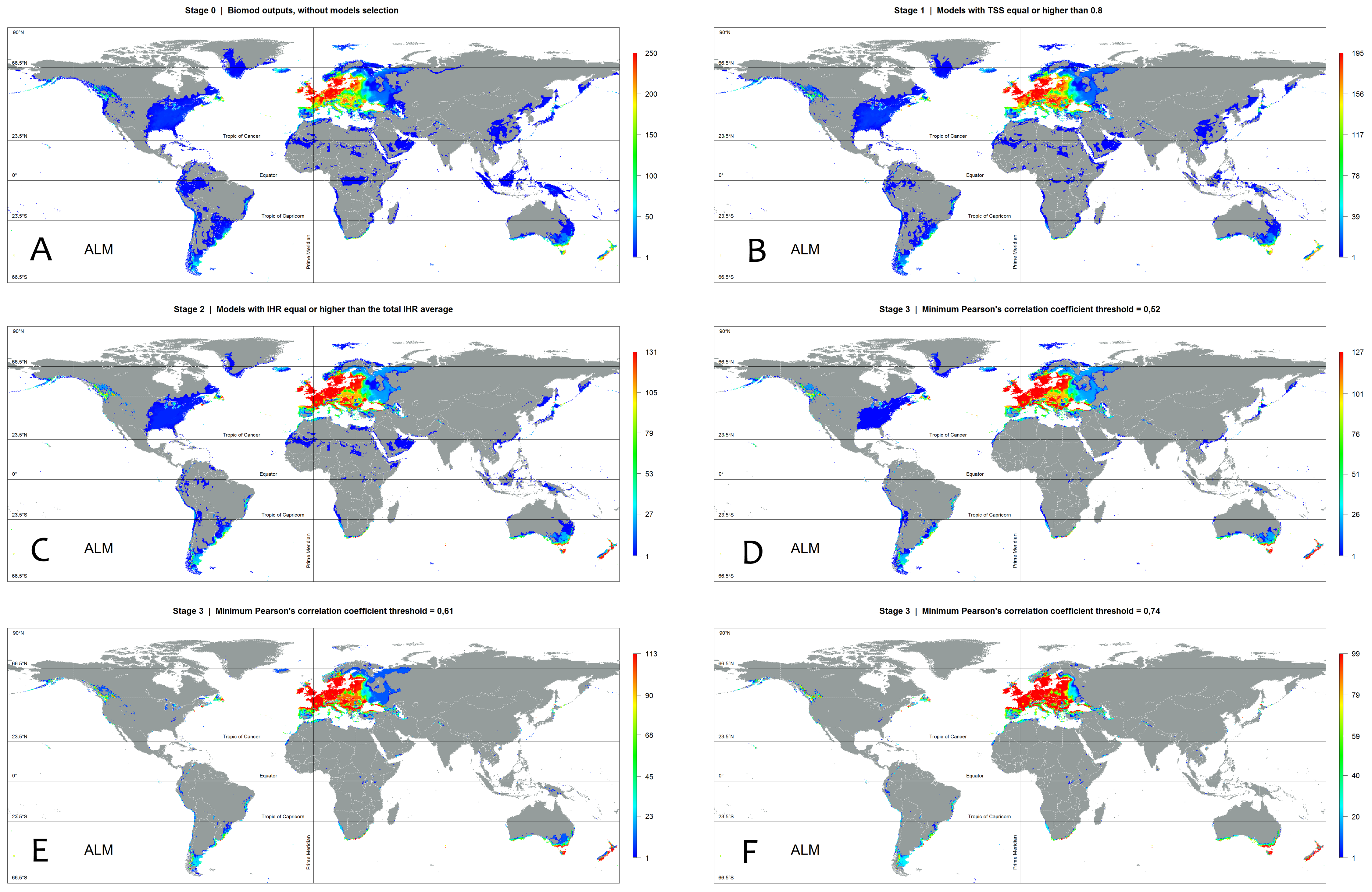

Supplement: S4 Fig — (TIF) [file pone.0148295.s004.tif]
