# Supplementary material for: Worldwide Alien Invasion: A Methodological Approach to Forecast the Potential Spread of a Highly Invasive Pollinator
Source: PLoS One. 2016 Feb 16;11(2):e0148295. doi: 10.1371/journal.pone.0148295 (PMC4755775; doi:10.1371/journal.pone.0148295)
Supplement: S1 File — (DOCX) [file pone.0148295.s005.docx]

**Data providers and bibliographical surveyed for *Bombus terrestris* native and invasive occurrences**

1 - Internet biodiversity data providers used for presences data survey and the amount of records after filtering by type of distribution (native or invasive; between brackets):

| GBIF - Global Biodiversity Information Facility (native: 8,614; invasive: 28). http://www.gbif.org/ |
| --- |
| DL - Discover Life (native: 1,225; invasive: 19). http://www.discoverlife.org/ |
| NBN - National Biodiversity Network (native: 946). http://www.nbn.org.uk/ |
| SeiyouStatus – See reference below: Seiyou Status, 2013 (invasive: 67).  http://www.seiyoubusters.com/seiyou/en/ |
| *Extracted from bibliography* – See references in S1.2 ( native: 467; invasive: 503). |

2 - Bibliographical References from which native and invasive occurrences data of *Bombus terrestris* were extracted.

2.1- *References used for occurrences data extraction that were also cited on text*:

Hopkins, 1914 [60]; Hingston and McQuillan, 1998 [99]; Ruz, 2002 [52]; Velthuis, 2002 [48]; Goulson and Hanley, 2004 [54]; Matsumura et al., 2004 [56]; AHGA, 2005 [102]; Inari et al., 2005 [57]; Torretta et al., 2006 [62]; Velthuis and van Doorn, 2006 [49]; Winter et al., 2006 [42]; Inoue et al., 2008 [63]; Schmid-Hempel et al., 2007 [104]; Ward, 2007 [13]; Kadoya et al., 2009 [15]; Kondo et al., 2009 [105]; Dafni et al., 2010 [40]; Howlett and Donovan, 2010 [58]; Kadoya and Washitani, 2010 [106]; Hingston et al., 2002 [51].

2.2- *References exclusively used for occurrences data extraction:*

Ban CM. Apoid hymenopterans (Megachilidae, Anthophoridae, Apidae) from Fagaras mountains area (Romania). Brvkenthal Acta Mvsei. 2006; I3: 107-112. (ISSN: 1842-2691). Available from: http://www.brukenthalmuseum.ro/pdf/BAM/BAM%20I%203.pdf

Ban-calefariu C, Sárospataki M. Contributions to the knowledge of Bombus and Psithyrus Genera (Apoidea: Apidae) in Romania. Travaux de Museum National d Historie Naturelle: Grigore Antipa. 2007; p239-258.

Beekman M, van Stratum P. Does the diapause experience of bumblebee queens Bombus terrestris affect colony characteristics? Ecol Entomol. 2000; 25:1-6.

Bloch G, Borst DW, Huang ZY, Robinson GE, Cnaani J, Hefetz A. Juvenile hormone titers, juvenile hormone biosynthesis, ovarian development and social environment in Bombus terrestris. J Ins Physiol. 2000; 46: 47-57.

Bosch M, Simon J, Rovira AM, Molero J, Blanche C. Pollination ecology of the pre-Pyrenean endemic Petrocoptis montsicciana (Cariophyllaceae): effects of population size. Biol J Linn Soc. 2002; 76:79-90.

Bourke AFG, Ratnieks FLW. Kin-selected conflict in the bumble-bee Bombus terrestris (Hymenoptera : Apidae) Proc R Soc Lond B. 2001; 268: 347-355.

Buide ML. Pollination ecology of Silene acutifolia (Caryophyllaceae): Floral traits variation and pollinator attraction. Ann Bot. 2006; 97: 289-297.

Buttermore RE, Pomeroy N, Hobson W, Semmens T, Hart R. Assessment of the genetic base of Tasmanian bumble bees (Bombus terrestris) for development as pollination agents. Journal of Apicultural Research. 1998;37: 23–25.

Buttermore, RE. Observations of successful Bombus terrestris (L) (Hymenoptera: Apidae) colonies in southern Tasmania. Australian Journal of Entomology. 1997;36:251-254.

Calzoni GL, Speranza A. Insect controlled pollination in Japanese plum (Prunus salicina Lindl). Sci Hort. 1998; 72: 227-237.

Coppée A, Terzo M, Valterova I, Rasmont P. Intraspecific variation of the cephalic labial gland secretions in Bombus terrestris (L) (Hymenoptera: Apidae). Chemistry & Biodiversity. 2008; 5(12): 2654-61.

Damerdji A, Djedid A. Contribuition à l'étude bioécologique de la faune du genêt (Calycotome spinosa L(Link)) dans la region de Tlemcen (Algérie). Mésogée. 2005; 61: 51-59.

Durieux PE. Rapport de recherche Etude des choix floraux des bourdons (Hymenoptera , Apidae) de la commune d'Eyne (France , Pyrénées-Orientales ), Université de Mons-Hainaut Service de Zoologie. 2000. Available from: http://zoologie.umh.ac.be/hymenoptera/biblio/Durieux_1999_Choix_floraux_Bourdons_Eyne_TFE.pdf

Duvoisin N, Baer B, Schmid-Hempel P. Sperm transfer and male competition in a bumblebee. Anim Behav. 1999; 58: 743-749.

Frehn E, Schwammberger KH. Social parasitism of Psithyrus vestalis in free-foraging colonies of Bombus terrestris (Hymenoptera : Apidae). Entomol Gen. 2001; 25: 103-105.

Furst MA, McMahon DP, Osborne JL, Paxton RJ, Brown, MJF. Disease associations between honeybees and bumblebees as a threat to wild pollinators. Nature. 2014; 506: 364-366.

Gadoum S, Iserbyt S, Michez D, Terzo M, Rasmont P. Les Abeilles sauvages du Parc naturel régional du Vexin français. Courrier Scientifique du Parc Naturel Régional du Vexin Français. 2005; 1: 28-33.

Genissel A, Aupinel P, Bressac C, Tasei JN, Chevrier C. Influence of pollen origin on performance of Bombus terrestris micro-colonies. Entomol Exp Appl. 2002; 104: 329-336.

Gosselin M, Iserbyt S, Rasmont P. Faunistique des bourdons (Hymenoptera: Apoidea ) de la vallée de Nohèdes (France, Pyrénées-Orientales ) et des zones limitrophes. Notes fauniques de Gembloux. 2007; 60 (1), 13-23.

Guitian J, Guitian P, Medrano M, Sanchez JM. Variation in floral morphology and individual fecundity in Erythronium dens-canis (Liliaceae). Ecography. 1999; 22: 708-714.

Hartfelder K, Cnaani J, Hefetz A. Caste-specific differences in ecdysteroid titers in early larval stages of the bumblebee Bombus terrestris. J Ins Physiol. 2000; 46: 1433-1439.

Herrera CM. Componentes del flujo génico en Lavandula latifolia medicus: polinización de dispersión de semillas. Anal J Bot Madrid. 1987; 44: 49-61.

Herrera CM, Cerda X, Garcia MB, Guitian J, Medrano M, Rey PJ, *et al.* Floral integration, phenotipic covariance structure and pollination variation in bumblebee-pollinated Helleborus foetidus. J Evol Biol. 2002; 15: 108-121.

Hirsch M, Pfaff S, Wolters V. The influence of matrix type on flower visitors of Centaurea jacea L. Agric Ecosyst Environ. 2003; 98: 331-337.

Ings TC, Ings NL, Chittka L, Rasmont P. A failed invasion? Commercially introduced pollinators in Southern France. Apidologie. 2009; 41(1): 1-13.

Ings TC, Ward NL, Chittka L. Can commercially imported bumble bees out-compete their native conspecifics? Journal of Applied Ecology. 2006; 43(5): 940-948.

Ings TC, Schikora J, Chittka L. Bumblebees, humble pollination or assiduous invaders? A population comparison of the foranging performance of Bombus terrestris. Oecologia. 2005; 144(3): 508-516.

Inoue MN, Yokoyama J. Competition for flower resources and nest sites between Bombus terrestris (L) and Japanese native bumblebees. Applied Entomology and Zoology. 2010; 45(1), 29-35.

Iserbyt S. La faune des bourdons (Hymenoptera?: Apidae) du Parc National des Pyrénées occidentales et des zones adjacentes. Ann Soc Entomol Fr. 2009; 45(2), 217-244.

Jablonski B. Agronomic and beekeeping value of short-tube populations of red clover (Trifolium pratense L). J Api Res. 2001; 45: 37-50.

Kanbe Y, Okada I, Yoneda M, Goka K, Tsuchida K. Interspecific mating of the introduced bumblebee Bombus terrestris and the native japanese bumblebee Bombus hypocrita sapporoensis results in inviable hybrids. Naturwissenschaften. 2008; 95(10):1003-1008.

Keasar T. The spatial distribution of non rewarding artificial flowers affects pollinator attraction. Anim Behav. 2000; 60: 639-646.

Keasar T, Motro U, Shur Y, Shmida A. Overnight memory retention of foraging skills by bumblebees is imperfect. Anim Behav. 1996; 52: 95-104.

Kreyer D, Oed A, Walther-Hellwig K, Frankl R. Are forests potential landscape barriers for foraging bumblebees? Landscape scale experiments with Bombus terrestris and Bombus pascuorum (Hymenoptera, Apidae). Biol Conserv. 2004; 116: 111-118.

Lopez-Vaamonde C, Koning JW, Jordan WC, Bourke AFG. No evidence that reproductive bumblebee workers reduce the production of new queens. Anim Behav. 2003; 66: 577-584.

Lopez-Vaamonde C, Koning JW, Jordan WC, Bourke AFG. A test of information use by reproductive bumblebee workers. Anim Behav. 2004;68:811-818.

Mahe G. Memo Pour L'atlas Des Bourdons Armoricains. 2012;1-11. Available from: http://www.atlashymenoptera.net/biblio/Memo2012_Atlas_Bombus_Armor.pdf.

Monsevièius V. Comparison of three methods of sampling wild bees (Hymenoptera, Apoidea) in Èepkeliai Nature Reserve (South Lithuania). Ekologija. 2004; 4:32-39.

Mtcsez D. Eco-éthologie des visiteurs de Lythrum salicaria L (Lythraceae) en Belgique. 2008; 10, 37-55.

Nagamitsu T, Yamagishi H, Kenta T, Inari N, Kato E. Competitive effects of the exotic Bombus terrestris on native bumble bees revealed by a field removal experiment. Population Ecology. 2009; 52(1), 123-136.

Olesen JM, Valido A. Floral morphology and pollen flow in the heterostylous species Pulmonaria obscura Dumort (Boraginaceaea). N Phytol. 1979; 82:757-767.

Oustrauskas H, Monsevièius V. Wild bees (Apoideat, Hymenoptera) caught with Green funnel traps in Lithuania. Acta Zool Lituan. 2002;12:191-196.

Pawlikowski T, Hirsch J. Bees (Hymenoptera: Apoidea) as indicators of xerisation processes in the lower Vistula Valley. Acta Zool Crac. 2002;45:321-333.

Petanidou T, Ellis WN. Pollinating fauna of a phyganic ecosystem: composition and diversity. Biodiversity Letters. 1993; 1:9-22.

Rasmont P. Bombus terrestris (L) (Hymenoptera, Apidae) dans les massif des Maures (France, Var), une generation d'Hiver? Bull Annal Soc R Belge d'Entomol. 1984; 120:359-363.

Rasmont P, Adamski A. Le bourdons de la Corse (Hymenoptera, Apoidea, Bombinae). Note Faun Gembloux. 1995; 31:3-87.

Rasmont P, Durieux E-A, Iserbijt S, Baracetti M. Why are there so many bumblebees species in Eyne (France, Pyrénée-Orientales, Cerdagne) IN: Sommeijer MJ, de Ruijter A (ed). Insect pollination in greenhouses: proceedings of the specialists' meeting held in Soesterberg, The Netherlands, Utrecht, Universiteit Utrecht, 2000. p220.

Rasmont P, Regali A, Ings T C, Lognay G, Baudart E, Marlier M, *et al.*Analysis of pollen and nectar of Arbutus unedo as a food source for Bombus terrestris (Hymenoptera: Apidae). Journal of Economic Entomology. 2005;98(3), 656-63.

Sarospataki M, Novak J, Molnar V. Distribution and relative abundance of bumble bee species (Bombus and Psithyrus) in Hungary. J, Api Res. 2003; 47:73-78.

Seiyou Status - Current status of invasive alien bumblebee Bombus terrestris by participatory monitoring in Hokkaido, Japan. 2013. Acessed: 2013-01-15. Available from: http://www.seiyoubusters.com/seiyou/en/
