# Supplementary material for: Worldwide Alien Invasion: A Methodological Approach to Forecast the Potential Spread of a Highly Invasive Pollinator
Source: PLoS One. 2016 Feb 16;11(2):e0148295. doi: 10.1371/journal.pone.0148295 (PMC4755775; doi:10.1371/journal.pone.0148295)
Supplement: S2 File — (DOCX) [file pone.0148295.s006.docx]

**Biomod2 Algorithms and Parameters**

Algorithms used in the modeling procedure: Artificial Neural Networks (ANN) [Ripley, 1996]; Classification Tree Analysis (CTA) [Breiman *et al*., 1984]; Generalized Boosted Models (GBM) [Ridgeway, 1999, Friedman, 2011]; Random Forests (RF) [Breiman, 2001]; Maximum Entropy (MAXENT) [Phillips *et al*., 2006]; Surface Range Envelops (SRE) [Busby, 1991]; Generalized Additive Models (GAM) [Hastie and Tibshirani, 1990]; Generalized Linear Models (GLM) [McCullagh and Nelder, 1989]; Multivariate Adaptive Regression Splines (MARS) [Friedman, 1991] and Flexible Discriminant Analysis (FDA) [Hastie and Tibshirani, 1994].

**-=-=-=-=-=-=-=-=-=-=-=-=-=-=-=-=-=-=-=-=-=-=** **'BIOMOD.Model.Options'** **-=-=-=-=-=-=-=-=-=-=-=-=-=-=-=-=-=-=-=-=-=-=**

GLM **=** list**(** type **=** **'quadratic'**,

interaction.level **=** 0,

myFormula **=** **NULL**,

test **=** **'AIC'**,

family **=** binomial**(**link **=** **'logit')**,

mustart **=** 0.5,

**control** **=** glm.control**(**epsilon **=** 1e**-**08, maxit **=** 50, trace **=** **FALSE)** **)**,

GBM **=** list**(** distribution **=** **'bernoulli'**,

n.trees **=** 2500,

interaction.depth **=** 7,

n.minobsinnode **=** 5,

shrinkage **=** 0.001,

bag.fraction **=** 0.5,

train.fraction **=** 1,

cv.folds **=** 3,

keep.data **=** **FALSE**,

verbose **=** **FALSE**,

perf.method **=** **'cv')**,

GAM **=** list**(** algo **=** **'GAM_mgcv'**,

type **=** **'s_smoother'**,

k **=** **-**1,

interaction.level **=** 0,

myFormula **=** **NULL**,

family **=** binomial**(**link **=** **'logit')**,

method **=** **'GCV.Cp'**,

optimizer **=** c**('outer'**,**'newton')**,

select **=** **FALSE**,

knots **=** **NULL**,

paraPen **=** **NULL**,

**control** **=** list**(**nthreads **=** 1, irls.reg **=** 0, epsilon **=** 1e**-**07, maxit **=** 100, trace **=** **FALSE**, mgcv.tol **=** 1e**-**07, mgcv.half **=** 15, rank.tol **=** 1.49011611938477e**-**08, nlm **=** list**(**ndigit**=**7, gradtol**=**1e**-**06, stepmax**=**2, steptol**=**1e**-**04, iterlim**=**200, check.analyticals**=**0**)**, optim **=** list**(**factr**=**1e**+**07**)**, newton **=** list**(**conv.tol**=**1e**-**06, maxNstep**=**5, maxSstep**=**2, maxHalf**=**30, use.svd**=**0**)**, outerPIsteps **=** 0, idLinksBases **=** **TRUE**, scalePenalty **=** **TRUE**, keepData **=** **FALSE)** **)**,

CTA **=** list**(** method **=** **'class'**,

parms **=** **'default'**,

cost **=** **NULL**,

**control** **=** list**(**xval **=** 5, minbucket **=** 5, minsplit **=** 5, cp **=** 0.001, maxdepth **=** 25**)** **)**,

ANN **=** list**(** NbCV **=** 5,

rang **=** 0.1,

maxit **=** 200**)**,

SRE **=** list**(** quant **=** 0.025**)**,

FDA **=** list**(** method **=** **'mars')**,

MARS **=** list**(** degree **=** 2, penalty **=** 2, thresh **=** 0.001, prune **=** **TRUE)**,

RF **=** list**(** do.classif **=** **TRUE**,

ntree **=** 500,

mtry **=** **'default'**,

nodesize **=** 5,

maxnodes **=** **NULL)**,

MAXENT **=** list**(** path_to_maxent.jar **=** **''**,

memory_allocated **=** 2048,

maximumiterations **=** 500,

visible **=** **FALSE**,

linear **=** **TRUE**,

quadratic **=** **TRUE**,

product **=** **TRUE**,

threshold **=** **TRUE**,

hinge **=** **TRUE**,

lq2lqptthreshold **=** 80,

l2lqthreshold **=** 10,

hingethreshold **=** 15,

beta_threshold **=** **-**1,

beta_categorical **=** **-**1,

beta_lqp **=** **-**1,

beta_hinge **=** **-**1,

defaultprevalence **=** 0.5**)**

**-=-=-=-=-=-=-=-=-=-=-=-=-=-=-=-=-=-=-=-=-=-=-=-=-=-=-=-=-=-=-=-=-=-=-=-=-=-=-=-=-=-=-=-=-=-=-=-=-=-=-=-=-=-=-=-=-=-=**

**References**

Breiman L, Friedman JH, Olshen RA, Stone CJ. Classification and regression trees. New York: Chapman and Hall; 1984.

Breiman L. Random Forests. Machine Learning. 2001; 45: 5-32. DOI:10.1023/a:1010933404324.

Busby JR. BIOCLIM - A bioclimate analysis and prediction system. In: Margule, CR, Austin MP (Eds). Nature Conservation: Cost Effective Biological Surveys and Data Analysis. Canberra, Australia: CSIRO; 1991. pp 64-68.

Friedman BJH. Reitz Lecture 29. 2011; p1189-1232. DOI: 10.1214/aos/1013203451.

Friedman J. Multivariate adaptive regression splines. Ann Stat. 1991; 19: 1-141.

Hastie T, Tibshirani R, Buja A. Flexible Discriminant Analysis by Optimal Scoring. JASA. 1994; p1255-1270.

Hastie TJ, Tibshirani RJ. Generalized Additive Models. Chapman & Hall: CRC Monographs on Statistics & Applied Probability. 1990.

McCullagh P, Nelder JA. Generalized Linear Models (2ed). Chapman & Hall/CRC Monographs on Statistics & Applied Probability. 1989.

Phillips SJ, Anderson RP, Schapire RE. Maximum entropy modeling of species geographic distributions. Ecological Modelling. 2006; 190: 231-259.

Ridgeway G. 2 Boosting for classification. Bernoulli. 1999; 172-181.

Ripley BD. Pattern recognition and neural networks. Cambridge, UK:Cambridge University Press. 1996.
