# Supplementary material for: Worldwide Alien Invasion: A Methodological Approach to Forecast the Potential Spread of a Highly Invasive Pollinator
Source: PLoS One. 2016 Feb 16;11(2):e0148295. doi: 10.1371/journal.pone.0148295 (PMC4755775; doi:10.1371/journal.pone.0148295)
Supplement: S1 Text — (DOCX) [file pone.0148295.s007.docx]

**Algorithms performance**

The low values of TSS achieved by SRE resulted in the exclusion of all its models early in Stage 1. This was expected because SRE presents a simple logic that uses extreme values (percentiles 0.025) of the environmental variables captured from the area where the presence records were located, and it uses the shared intervals of all variables (between minimum and maximum limits) to delineate the suitable spatial shape [Thuiller *et al*., 2009, Thuiller *et al*., 2013]. The large dimension of inputted data into the modeling procedure led to extremely restrictive results, predicting very small-size suitable areas and consequently, low TSS values and the worst algorithm result.

The classification method used by FDA is influenced by the amount of input data, as very large dimensions (with high variability) may lead to reduced classification performance [Hastie *et al*., 1994]. This likely influenced the performance of FDA, which was also poor. The suitability shape of predicted areas among FDA models was very discordant, especially at the borders, and almost the entire shared suitability area was restricted to the range covered by the inputted native *Bombus terrestris* (hereinafter referred as Bt) presence records. In general, FDA models exhibited evident underfitting compared to other models (and algorithms); nevertheless, two of its models passed Stages 1, 2 and the first PCC (0.52) of Stage 3.

In spite of the majority of MARS-generated models have reached TSS values slightly above the minimum threshold, they predicted a large and central unsuitable area covered by native Bt range. The shape of this unsuitable area, surrounded by suitable ones, diverged from almost all other models. Thus, across the PCC range, MARS dissimilarity resulted in the reduction of models at higher PCC values until total exclusion after PCC 0.61 (Stage 3). MARS and FDA models shared some predictive similarities, possibly related to the fact that FDA uses MARS in its regression component. However, based solely on our results, MARS alone performed better than FDA.

Some algorithms, namely, ANN, CTA and RF, were strongly affected by Stage 2, which reduced the number of generated models. These exclusions suggest that the predictive logics used by these algorithms could be considered good for detecting coherent suitable areas within or near the presence records used to train the models, based on the TSS. However, they also suggest that these algorithms are poor at extrapolating predictions over areas more distant from the training data; thus, these algorithms presented low extrapolative ability.

Almost all predictions from ANN showed clear indications of overfitting and presented many statistical artifacts. For example, ANN indicated Bt suitability in the extreme arid regions of the Sahara and the extremely humid regions of Amazonian forest. In addition, the large susceptible area predicted in eastern North America in the final ensemble model resulted from a unique ANN model that remained through Stage 3. Based in the Invasive Hit Rate (IHR), almost no ANN models were able to extrapolate suitability predictions. Moreover, ANN showed highly divergent predictions among its own models (as expected considering its logic; [Thuiller *et al*., 2009, Thuiller *et al*., 2013] but also divergent predictions compared to the models of other algorithms. Despite these properties, 22 ANN models passed Stage 1. This finding suggests a possible inadequacy in the overall number of random generated pseudo-absences, resulting in inefficiency in the specificity component of TSS. We used a large number of pseudo-absences: for each pseudo-absence partitioning, we used ten times the number of training presences [Chefaoui and Lobo, 2008]. However, TSS seems unable to detect the overfitted models generated by ANN. CTA models also exhibited divergent suitability predictions among its models but, in contrast to ANN, the predicted suitable areas were relatively small compared to all other predictions. However, some CTA models exhibited convergence with others, reaching higher PCC thresholds.

RF performance was particularly intriguing. Our results showed that despite the high TSS values obtained by RF, no model was selected past Stage 2.This result suggests some limitation of TSS in evaluating the extrapolative capacity of the models. While we defined a restriction zone in the range of the native Bt distribution, avoiding pseudo-absences in this area, we did not define any restriction to pseudo-absences on known invaded areas. We took this approach because we wanted the algorithms to be able to accurately detect suitability covering the invasive records without interventions in the modeling procedure relative to the invasive dataset. However, this aspect apparently reduced the efficiency of the TSS specificity component, hindering the capacity of TSS to correctly evaluate predictions in areas distant from the inputted training data. However, we emphasize that each algorithm used the same combination of five pseudo-absence datasets and five presence partitioning for testing and training; thus, there is no bias related to conditions of the input data. Overall, our results suggest that the exclusive use of TSS as a metric to evaluate the accuracy of models may be insufficient when a good suitability extrapolation is required. We demonstrated that the use of a “validation dataset” (Stage 2) can improve the evaluation procedure and validate the selection of models with high extrapolative accuracy.

The algorithms that yielded the best results were GAM, GLM, MAXENT and GBM. GAM and GLM have been described as having increased accuracy with increasing numbers of presence records used to train the model [Aguirre-Gutiérrez *et al*., 2013]. The highest performance detected here by both algorithms was possibly related to the extremely large number of Bt presence records used. MAXENT and GBM also performed very well; both algorithms use more complex logics than the others, and MAXENT is known to progressively increase the mathematical complexity of the model with increasing available data [Elith *et al*., 2011, Aguirre-Gutiérrez *et al*., 2013]. Moreover, the highest performance of both algorithms is reached in scale-reduced species distribution ranges and when using small training datasets [Aguirre-Gutiérrez *et al*., 2013]. It is likely that these aspects explain why MAXENT and GBM performed slightly worse than GAM and GLM in our study. Together, these four algorithms provided the majority of models contributing to each Stage 3 model.

The high quality of the predictions of these four algorithms is apparent in the large number of models that remained after the three-stage process. Moreover, the large number of shared predictions among these four algorithms and the large number of shared predictions among the set of models generated by each suggest a high level of coherence and robust mathematical logics.

Despite some minimal variation between datasets (five PAs and native presence partitioning), the models yielded very similar and accurate results. This is also apparent when comparing algorithms, as each one uses particular mathematical logics to predict suitability and generate models, but they can nonetheless yield similar and accurate results. Therefore, our framework helped us to detect and filter divergent models from algorithms whose results were unreliable.

**References**

Aguirre-Gutiérrez J, Carvalheiro LG, Polce C, Van Loon EE, Raes N, Reemer M, *et al*. Fit-for-Purpose: Species Distribution Model Performance Depends on Evaluation Criteria - Dutch Hoverflies as a Case Study. PloS one. 2013; 8 (5), e63708.

Chefaoui RM, Lobo JM. Assessing the effects of pseudo-absences on predictive distribution model performance. Ecological Modelling. 2008; V210, 4: 478-486.

Elith J, Phillips SJ, Hastie T, Dudík M, Chee YE, Yates, CJ. A statistical explanation of MaxEnt for ecologists. Diversity and Distributions, 2011; 17: 43-57.

Hastie T, Tibshirani R, Buja A. Flexible Discriminant Analysis by Optimal Scoring. JASA. 1994; p1255-1270.

Thuiller W, Georges D, Engler R. Ensemble platform for species distribution modeling - Package "biomod2". 2013. Available from: http://cran.r-project.org/web/packages/biomod2/biomod2.pdf

Thuiller W, Lafourcade B, Engler R, Araújo MB. BIOMOD - A platform for ensemble forecasting of species distributions. Ecography. 2009; 32: 369-373.
